# The *Mbd1*-*Atf7ip*-*Setdb1* pathway contributes to the maintenance of X chromosome inactivation

**DOI:** 10.1186/1756-8935-7-12

**Published:** 2014-06-26

**Authors:** Alissa Minkovsky, Anna Sahakyan, Elyse Rankin-Gee, Giancarlo Bonora, Sanjeet Patel, Kathrin Plath

**Affiliations:** 1 David Geffen School of Medicine, Department of Biological Chemistry, Jonsson Comprehensive Cancer Center, Molecular Biology Institute, Eli and Edythe Broad Center of Regenerative Medicine and Stem Cell Research, University of California, Los Angeles, CA 90095, USA

**Keywords:** X chromosome inactivation, *Xist*, H3K9 methylation, Atf7ip, Mcaf1, Mbd1, Setdb1

## Abstract

**Background:**

X chromosome inactivation (XCI) is a developmental program of heterochromatin formation that initiates during early female mammalian embryonic development and is maintained through a lifetime of cell divisions in somatic cells. Despite identification of the crucial long non-coding RNA *Xist* and involvement of specific chromatin modifiers in the establishment and maintenance of the heterochromatin of the inactive X chromosome (Xi), interference with known pathways only partially reactivates the Xi once silencing has been established. Here, we studied ATF7IP (MCAF1), a protein previously characterized to coordinate DNA methylation and histone H3K9 methylation through interactions with the methyl-DNA binding protein MBD1 and the histone H3K9 methyltransferase SETDB1, as a candidate maintenance factor of the Xi.

**Results:**

We found that siRNA-mediated knockdown of *Atf7ip* in mouse embryonic fibroblasts (MEFs) induces the activation of silenced reporter genes on the Xi in a low number of cells. Additional inhibition of two pathways known to contribute to Xi maintenance, DNA methylation and *Xist* RNA coating of the X chromosome, strongly increased the number of cells expressing Xi-linked genes upon *Atf7ip* knockdown. Despite its functional importance in Xi maintenance, ATF7IP does not accumulate on the Xi in MEFs or differentiating mouse embryonic stem cells. However, we found that depletion of two known repressive biochemical interactors of ATF7IP, MBD1 and SETDB1, but not of other unrelated H3K9 methyltransferases, also induces the activation of an Xi-linked reporter in MEFs.

**Conclusions:**

Together, these data indicate that *Atf7ip* acts in a synergistic fashion with DNA methylation and *Xist* RNA to maintain the silent state of the Xi in somatic cells, and that *Mbd1* and *Setdb1*, similar to *Atf7ip*, play a functional role in Xi silencing. We therefore propose that ATF7IP links DNA methylation on the Xi to SETDB1-mediated H3K9 trimethylation via its interaction with MBD1, and that this function is a crucial feature of the stable silencing of the Xi in female mammalian cells.

## Background

X chromosome inactivation (XCI) is an X chromosome-wide process of gene silencing in female mammalian cells that is a mechanism for dosage compensation of X-linked gene products between the sexes. Due to the random nature of XCI, normal adult female mammalian organisms have a roughly equal distribution of cells with an inactive X chromosome (Xi) of maternal or paternal origin [1]. Random XCI is initiated in early embryonic development in pluripotent epiblast cells shortly after the blastocyst implants. Once established, XCI becomes remarkably stable and is maintained for the lifetime of somatic cells, but is reversed in the cells that give rise to the germline [2-5]. Experimentally, the initiation of XCI can be recapitulated in differentiating female mouse embryonic stem cells (ESCs). Notably, despite its extreme stability in differentiated cells *in vivo*, the reactivation of the Xi can be achieved experimentally by induction of the pluripotent state in somatic cells either through transcription factor-induced reprogramming to induced pluripotent stem cells (iPSCs), cell fusion with ESCs, or somatic cell nuclear transfer [6-8].

XCI begins with the upregulation and spread of the long non-coding RNA *Xist* on the future Xi [2-5]. *Xist*, itself encoded on the X chromosome, is absolutely required for the initiation of XCI and its coating of the chromosome initiates a cascade of silencing events including the exclusion of RNA polymerase II from the *Xist* RNA*-*coated domain, the gain of histone H3K9me2, loss of active chromatin marks such as histone H3K4 methylation and histone acetylation, the recruitment of polycomb repressive complexes (PRC) 1 and 2 and deposition of their respective histone marks H2AK119ub1 and H3K27me3, and the gain of H4K20me1 [2-5]. After the establishment of the Xi, XCI transitions to the maintenance phase in which the repressed state of the X chromosome is stabilized and permanently locked in. The maintenance phase is marked by incorporation of the histone variant macroH2A, accumulation of promoter CpG methylation, and transition to late replication in S-phase [9-12].

Many of the protein factors implicated in XCI were identified based on their nuclear enrichment on the Xi by immunofluorescence [13]. Notably, one of these Xi-enriched proteins is the matrix protein hnRNP U, which has been shown to be required for the *Xist* RNA coating of the Xi and the initiation of X-inactivation [14]. Yet, various chromatin modifiers implicated in XCI, including Eed (the structural subunit of PRC2), Ring1b (the E3 ligase of PRC1), G9a (a histone methyltransferase mediating H3K9 methylation), and Dnmt3a/b (the *de novo* DNA methyltransferases) were found to be dispensable for both initiation and maintenance of X chromosome silencing in mice and in *in vitro* cell culture systems [15-18]. Therefore, the exact role for some of these factors in random XCI still remains unclear.

Interestingly, in the course of XCI initiation, the cascade of chromatin and transcriptional changes depends on continued *Xist* expression and remains reversible upon experimentally induced *Xist* shutdown [19]. In contrast, the maintenance phase of XCI is characterized by almost complete resistance of the Xi to reactivation upon *Xist* deletion [19,20]. To explain this switch in *Xist* dependence with XCI phase, studies in differentiated female cells have described synergism between *Xist* RNA, DNA methylation, histone variants, and histone hypoacetylation in maintaining XCI [20-22]. For instance, assaying primary mouse embryonic fibroblasts (MEFs) harboring a GFP reporter on the Xi that is subject to X-inactivation, showed reactivation in approximately 11% of cells 13 days after simultaneous deletion of *Xist* and *Dnmt1*[20]. The contribution of *Xist* to silencing is considerably smaller than that of *Dnmt1* as *Xist* deletion alone only doubled the low ‘spontaneous’ background rate of reactivation to 0.05%, while *Dnmt1* deletion alone led to approximately 5% reactivation [20]. Thus, multiple epigenetic layers act together to maintain the silenced state of the Xi and *Xist* retains some role in gene silencing in the maintenance phase that is appreciated only when other repressive modifications are inhibited. Another instance of this cooperative functional role of repressive modifications in XCI involves the histone variant macroH2A as well as Cullin3 and SPOP (both members of an E3 ligase complex that ubiquitinates macroH2A) [21,22]. Knockdown of any one of these three proteins alone does not induce activation of the Xi-linked GFP reporter, but rates of activation increase when knockdown is sensitized by a DNA demethylating agent and a histone deacetylase inhibitor [21,22].

In summary, these studies demonstrate that the Xi in somatic cells is relatively resistant to reactivation by interference with single known factors and that seemingly distinct silencing mechanisms act in a combinatorial fashion to ‘lock-in’ the heterochromatin state. Notably, among the chromatin mechanisms tested for a functional role in Xi-maintenance, DNA methylation so far appears to be the most critical pathway for maintaining the silent state of the Xi in differentiated cells [20]. However, one fundamental repressive mechanism, histone H3K9 methylation, also enriches on the Xi but its importance in XCI has not yet been clearly established, raising the question of whether this methylation mark and the enzymes involved in its deposition functionally contribute to Xi-maintenance [23-27]. Furthermore, since in many developmental systems DNA methylation and histone H3K9 methylation pathways can be dependent on one another, an interesting question is whether these two chromatin regulatory mechanisms would also be linked on the Xi [28].

In this study, we focused on these questions by dissecting the functional contribution of the nuclear protein ATF7IP (also known as MCAF1 for Mbd-1 Chromatin Associated Factor 1) to XCI. We chose to test the function of ATF7IP in XCI for two reasons. First, though described to function as both a transcriptional activator and repressor, ATF7IP is a particularly interesting candidate factor for a regulator of XCI because in its repressive context, it has been shown to mediate MBD1-dependent transcriptional silencing through the recruitment and catalytic activation of the histone H3K9 methyltransferase SETDB1 [29-31]. Since MBD1 (methyl-CpG DNA Binding Domain Protein 1) binds methylated DNA, this function of ATF7IP could thereby bridge the DNA methylation and histone H3K9 methylation pathways on the Xi [32]. Second, ATF7IP is implicated to interact with CDYL, a chromodomain-containing transcriptional co-repressor that is recruited to the Xi by H3K9me2, as ATF7IP was recently identified by mass spectrometry in a CDYL pulldown in undifferentiated and differentiating mouse ESCs [27].

Our study identifies ATF7IP and its previously described biochemical interactors MBD1 and SETDB1 as regulators of the Xi in differentiated cells. We reveal this function of *Atf7ip*, *Mbd1*, and *Setdb1* in a ‘sensitized’ approach, where we add low concentrations of 5-aza-2’-dC to Xi-reactivation assays with the rationale of destabilizing DNA methylation and therefore XCI, so that interference with candidate gene function would increase rates of Xi-reactivation in somatic cells.

## Results and discussion

### Depletion of *Atf7ip* leads to Xi-reactivation in somatic cells

Recently, the chromodomain-containing transcriptional repressor CDYL was found to associate with the Xi, requiring the induction of differentiation and both histone H3K9me2 and H3K27me3 for its Xi-enrichment [27]. Based on its biochemical interaction with the H3K9 methyltransferase G9A, it was proposed that CDYL anchors G9A to facilitate the propagation and maintenance of the H3K9me2 mark on the Xi and ensure the perpetuation of the H3K9me2 on the Xi during S-phase [27]. Based on the timing of Xi association and its independence of the silencing domain of *Xist*, it was suggested that CDYL-regulated processes play a role in the maintenance of XCI rather than the initiation of X-inactivation. Interestingly, Heard and colleagues also found ATF7IP (MCAF1) and its homolog ATF7IP2 (MCAF2) as one of 20 high-confidence proteins interacting with CDYL in both undifferentiated and differentiating mouse ESCs [27], leading us to test the hypothesis that ATF7IP, a protein known to link the DNA methylation and H3K9 methylation pathways in autosomal gene silencing contexts, could also function in XCI.

**Figure 1:**
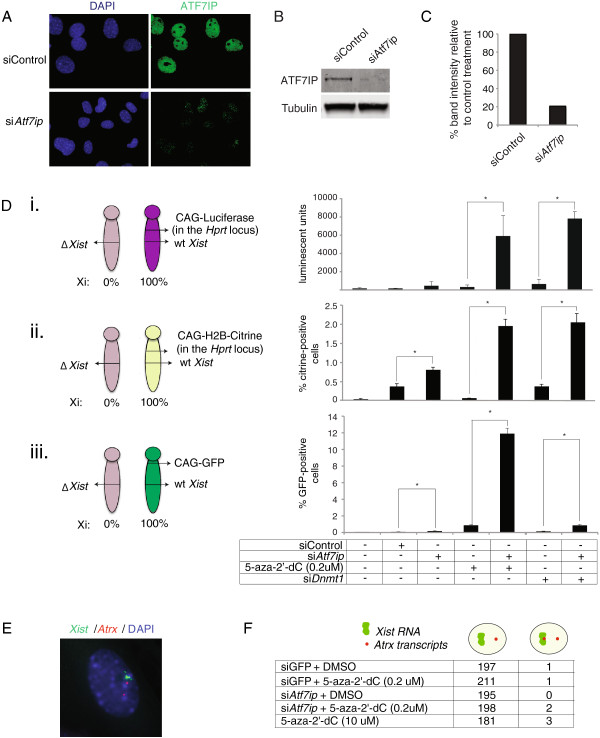
***Atf7ip *is required for Xi maintenance and acts synergistically with DNA methylation. (A)** Representative immunostaining images of MEFs treated with control knockdown (in this case targeting a sequence within GFP) or *Atf7ip* knockdown for 72 h and stained for ATF7IP (green). Nuclei were detected with DAPI stain. **(B)** Western blot of cells treated as in (A) with antibody against ATF7IP (band size approximately 220 kDa), with alpha-tubulin loading control. **(C)** Quantification of band intensity of the western blot shown in **(B)**, normalized to alpha-tubulin loading control. **(D)** (Left) Schematic of the X chromosomes in female reporter MEFs carrying (i) the Xi-linked luciferase (Xi^CAG-Luciferase^Xa^ΔXist^ MEFs), (ii) H2B-Citrine (Xi^CAG-H2B-Ctirine^Xa^ΔXist^ MEFs), or (iii) GFP (Xi^CAG-GFP^Xa^ΔXist^ MEFs). (Right) Graphs summarizing the reporter assays for respective MEFs treated with control siRNA treatment (siScramble), siRNAs targeting *Atf7ip* and *Dnmt1*, and/or 5-aza-2’-dC (0.2 uM) (also referred to as low concentration 5-aza-2’-dC) as indicated, for 72 h. Error bars indicate standard deviation of raw ALU values (for luciferase) or fluorescent cell count (for GFP and H2B-citrine) from three individual wells with the same treatment from one representative experiment. * = *P* <0.01 by Student’s T-test. **(E)** RNA FISH for *Xist* and the X-linked gene *Atrx* in MEFs 72 h after addition of 0.2 um (low) 5-aza-2’-dC and transfection of siRNAs targeting *Atf7ip. Atrx* pinpoints of transcript signal mark the site of nascent transcription. Representative image for a cell displaying expression of *Atrx* from the Xi, with two pinpoints for *Atrx*, one associated with the *Xist* RNA cloud. **(F)** Summary of counts of nuclei analyzed by RNA FISH for *Atrx* and *Xist* RNA, with the indicated FISH patterns.

To begin exploring the role of ATF7IP in XCI, we first investigated the consequences of *Atf7ip* depletion on the maintenance of the silent state of the Xi in somatic cells. Knockdown of *Atf7ip* in MEFs reduced protein levels by approximately 75% and dramatically decreased the global nuclear protein signal as assayed by immunofluoresence (Figure 1A-C, Additional file 1: Figure S1A/B), while transcript levels were decreased by about 50% (Additional file 1: Figure S1C). The specificity of the antibody directed against ATF7IP and the specific targeting of *Atf7ip* by the siRNA were validated further by immunostaining and knockdown, respectively, of ectopically expressed FLAG-tagged ATF7IP. Specifically, female MEFs transduced with a retroviral vector expressing FLAG-tagged ATF7IP showed a similar nuclear distribution of the FLAG-tagged protein and the endogenous protein in immunostaining experiments (Additional file 1: Figure S1D). Furthermore, retrovirally encoded FLAG-ATF7IP was expressed at six-fold higher levels than endogenous *Atf7ip* as determined by RT-PCR, and the *Atf7ip*-targeting siRNA was able to target the exogenous product and reduce its transcript levels by about 50% (Additional file 1: Figure S1E).

To monitor the functional effect of *Atf7ip* knockdown on the maintenance of the silent state of the Xi, we developed a new Xi-linked reporter that is highly sensitive for Xi-reactivation. Specifically, the firefly luciferase open reading frame under control of the constitutive CAG promoter was targeted into the *Hprt* locus on the single X chromosome in male mouse ESCs using Flp-mediated recombination, an approach similar to one described previously [33] (Additional file 2: Figure S2). Correct targeting was confirmed by southern blotting (data not shown). Subsequently, transgenic mice were generated by blastocyst injection of a targeted ESC clone. Upon germline transmission, skewing of XCI to the luciferase-bearing X chromosome was ensured by breeding male reporter mice with female mice heterozygous for an *Xist* knockout (ΔXist) allele [34]. It has been previously demonstrated that the loss of *Xist* on one X chromosome in female mice leads to inactivation of the wild-type X chromosome in 100% of the cells, thereby resulting in the *Xist*-deleted X chromosome remaining active (the Xa) in all cells [35].

As expected, Xi^CAG-Luciferase^Xa^ΔXist^ MEFs derived from these animals (carrying the luciferase reporter on the Xi in all cells) did not show luciferase expression in normal culture conditions (Additional file 3: Figure S3Ai, untreated condition). By contrast, female MEFs carrying wild-type *Xist* on both X chromosomes and heterozygous for CAG-luciferase in the *Hprt* locus displayed strong luciferase expression due to random XCI (Additional file 3: Figure S3Aii). These results indicate that our new reporter is subject to developmental XCI and becomes efficiently silenced when located on the Xi. In agreement with this finding, the luciferase reporter gene including its promoter was found to be highly methylated on the Xi in Xi^CAG-Luciferase^Xa^ΔXist^ MEFs as determined by reduced-representation bisulfite sequencing (Additional file 4: Figure S4A, B). Luciferase expression in Xi^CAG-Luciferase^Xa^ΔXist^ MEFs increased when the DNA methylation machinery was impaired by either depleting *Dnmt1* with siRNAs or treating cells with the demethylating agent 5-aza-2’-dC (Figure 1Di, Additional file 3: Figure S3Ai). Accordingly, levels of DNA methylation at the luciferase reporter decreased upon treatment with a high concentration of 5-aza-2’-dC (10 uM) (Additional file 4: Figure S4B, C). Thus, the luciferase reporter in the *Hprt* locus on the Xi can be activated efficiently when a known pathway of Xi maintenance is impaired. Therefore, we used these fibroblasts to examine the role of *Atf7ip* in XCI.

We found that *Atf7ip* knockdown alone yielded weak activation of the luciferase reporter in Xi^CAG-Luciferase^Xa^ΔXist^ MEFs after three days of knockdown (at 72 h of treatment) (Figure 1Di). While this effect was often seen, it did not consistently pass significance tests. Since it is known that various repressive mechanisms synergistically act to maintain the silent state of the Xi, we reasoned that combining *Atf7ip* knockdown with conditions of DNA demethylation may unmask an effect of *Atf7ip* depletion [20]. We first used the demethylating agent 5-aza-2’-dC, which inhibits DNMT1 and impedes propagation of DNA methylation through cell division [36]. On its own, 5-aza-2’-dC had a dose-dependent activity as increasing concentration induced higher rates of Xi-reactivation based on the luciferase reporter assay (Additional file 3: Figure S3Ai). In our *Atf7ip* knockdown experiments (Figure 1Di), we chose to add 0.2 uM 5-aza-2’-dC, since that amount of 5-aza-2’-dC induced only very little luciferase activity on its own (Additional file 3: Figure S3Ai). When combined with this low level of 5-aza-2’-dC, luciferase reporter reactivation due to *Atf7ip* knockdown was reproducibly enhanced up to 50-fold (Figure 1Di). To confirm that the Xi-reactivation effect of 5-aza-2’-dC treatment on *Atf7ip* knockdown was indeed due to changes in DNA methylation and not through potential off-target effects of 5-aza-2’-dC, we also depleted *Dnmt1*, the main enzyme responsible for the maintenance of DNA methylation, with siRNAs in place of 5-aza-2’-dC treatment (Additional file 3: Figure S3B). Again, we observed strong enhancement in reporter activation when *Atf7ip* was depleted concurrently with *Dnmt1,* while knockdown of either *Atf7ip* or *Dnmt1* alone induced very low levels of luciferase activity (Figure 1Di). Addition of low levels of 5-aza-2’-dC did not significantly alter the efficacy of Atf7ip knockdown as assayed by western blot (compare Figure 1B, C to Additional file 1: Figure S1A, B). Therefore, we conclude that depletion of *Atf7ip* on its own has a limited effect on the Xi, but greatly induces Xi-reactivation when the DNA methylation maintenance pathway is impaired concomitantly. These data reveal a synergistic effect on the Xi between ATF7IP and the DNA methylation pathway.

We recapitulated these results with MEFs carrying another newly generated fluorescence reporter (histone H2B fused to citrine - H2B-citrine) in the same locus as the luciferase reporter (*Hprt*) (Figure 1Dii), and with MEFs carrying a previously published randomly integrated GFP transgene near the centromere on the X chromosome (Figure 1Diii) [20,37], allowing us to quantify the number of cells with Xi-reactivation by FACS. In both cases, depletion of only *Atf7ip* induced reporter activation in a low but significantly higher number of cells than control treatments. Inhibition of DNA methylation either with a low concentration 5-aza-2’-dC or by knockdown of *Dnmt1* dramatically increased this effect. Taken together, the activity of *Atf7ip* depletion against three Xi reporters strongly supports a role for *Atf7ip* in the maintenance of the Xi.

We noted that reactivation in response to knockdown of *Atf7ip* and DNA methylation inhibition occurred in approximately 12% of GFP-reporter MEFs and in only approximately 2% of H2B-citrine Xi-reporter MEFs based on flow cytometry measurements (Figure 1Dii/iii), which is potentially due to different levels of DNA methylation, other chromatin marks, and/or their integration sites. Notably, we also observed that the X-GFP reporter, when located on the Xa, was expressed at a uniformly high intensity across the cell population while, upon reactivation from the Xi, the GFP intensity was much less uniform and ranged from low to high expression levels (Additional file 5: Figure S5A, compare blue cell population). The H2B-citrine Xi-reporter displayed a similar gradient of reactivation levels upon treatment with *Atf7ip* knockdown and 5-aza-2’-dC (Additional file 5: Figure S5B). We believe that this gradient in fluorescence intensity under reactivation conditions is a reflection of partial reactivation of the Xi-linked reporter transgenes that is different in each cell, likely owing to incomplete demethylation of the reporters and the interaction with persistent silencing chromatin marks.

Given the different extent of reactivation observed with our two fluorescent reporter MEF lines and the cell-to-cell heterogeneity in Xi fluorescent reporter intensity, we next set out to determine whether *Atf7ip* knockdown also influenced the expression of an endogenously encoded X-linked gene that is subject to XCI. Specifically, we monitored the activation of *Atrx* by RNA fluorescence *in situ* hybridization (FISH) relative to the *Xist* RNA cloud under reactivation treatment conditions. In cells of normal ploidy, we noticed rare nuclei with two spots of nascent *Atrx* transcripts with one of the two spots being in proximity of the *Xist* RNA cloud (Figure 1E). Quantification of the FISH data suggests a rate of *Atrx* reactivation of 0.5% to 1.0% in response to high concentration 5-aza-2’-dC or low 5-aza-2’-dC combined with *Atf7ip* knockdown (Figure 1F), which contrasts the rates of Xi-reactivation of 2% to 12% that was observed with the fluorescent reporters. However, we note that the rare numbers of cells with *Atrx* reactivation on the Xi were not consistently observed across independent treatments and FISH counts. While the FISH data show a trend towards reactivation of an endogenous gene on the Xi in response to *Atf7ip* knockdown, we interpret the lower magnitude of reactivation as compared to the Xi-reporter approach as stemming from poorer sensitivity of FISH compared to reporter assays. We believe that almost complete reactivation of *Atrx* would be required to allow the detection of a *Atrx* signal at the site of transcription on the Xi with our FISH methodology, but is likely not attained upon our treatments [38]. This conclusion is supported by the limited extent of reactivation of our fluorescent reporters described above (Additional file 5: Figure S5).

### ATF7IP does not accumulate on the Xi

**Figure 2:**
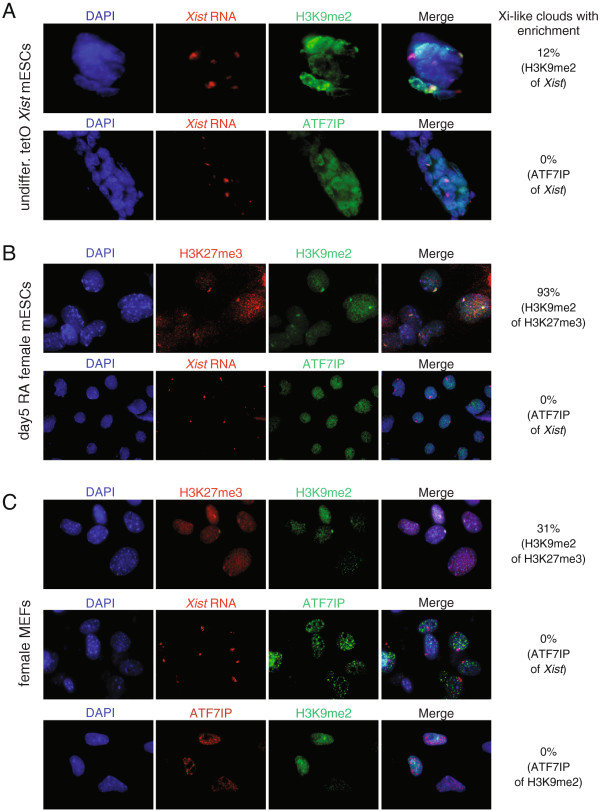
**Nuclear localization of ATF7IP relative to *Xist *RNA, H3K27me3, and H3K9me2 during the initiation and maintenance phases of XCI. (A)** Undifferentiated male mouse ESCs carrying a tet-inducible promoter at the endogenous *Xist* locus were analyzed by FISH for *Xist* RNA (red) and immunostaining for H3K9me2 and ATF7IP, respectively (green), 21 h after induction of *Xist* expression with doxycycline. DAPI was used to detect nuclei. Representative images are shown and the numbers on the right of the merged images indicates the percentage of cells (n = 100 per sample) with H3K9me2 or ATF7IP enrichment within the *Xist* RNA-demarcated X chromosome. **(B)** Similar to **(A)**, except that female mouse ESCs after 5 days of retinoic acid (RA) differentiation were analyzed by H3K27me3/H3K9me2 co-immunostaining or *Xist* RNA FISH in combination with ATF7IP immunostaining. **(C)** Similar to **(A)**, except that female MEFs were stained for H3K9me2, H3K27me3, ATF7IP, and *Xist* RNA in the indicated combinations.

Since our results indicated that *Atf7ip* is crucial for the maintenance of the silent state of the Xi in somatic cells, particularly when DNA methylation is impaired, we next wanted to determine whether ATF7IP enriches on the Xi in the initiation and/or maintenance phases of XCI, employing a combined immunostaining/FISH approach. As controls, we also detected H3K9me2 and H3K27me3, which have very predictable Xi-enrichment patterns in various phases of XCI [27,39,40]. To assess the initiation phase, we used previously described male ESCs with a doxycycline-inducible *Xist* allele [41], and combined the immunostaining for ATF7IP with FISH for *Xist* RNA. We found that these cells lacked an accumulation of ATF7IP on the *Xist* RNA-coated chromosome after 21 h of doxycycline treatment (Figure 2A). In agreement with previous reports, in the majority of these *Xist*-expressing undifferentiated ESCs, *Xist* RNA coating of the X chromosome was accompanied by a strong H3K27me3 enrichment, and a more infrequent and less prominent accumulation of H3K9me2 (Figure 2A, and data not shown) [27,39,40]. These results indicate that ATF7IP does not accumulate on the Xi during the initiation phase of XCI.

We next evaluated whether ATF7IP accumulates at later stages of XCI, by analyzing its nuclear pattern in female differentiating ESCs and female MEFs. In female ESCs induced to differentiate with retinoic acid for five days, the majority of H3K27me3/*Xist* RNA-demarcated X chromosomes was coated with H3K9me2 (as described in [27]), yet again no ATF7IP enrichment was observed (Figure 2B). Similarly, in female MEFs, where a smaller proportion of H3K27me3-demarcated inactive X chromosomes have H3K9me2 enrichment, ATF7IP displayed a nuclear staining pattern without any specific Xi enrichment (Figure 2C).

In summary, these data indicate that ATF7IP does not specifically accumulate on the Xi in any of the tested cellular systems, however this does not exclude the possibility that ATF7IP is recruited to the Xi. It is possible that ATF7IP can fulfill its critical function in the maintenance phase of XCI as part of a dynamic interaction with the Xi that cannot be captured by immunostaining approaches. Alternatively, these data may suggest that ATF7IP functions on the Xi without being actively recruited to the Xi by *Xist* RNA.

### ATF7IP’s function in XCI is independent of *Xist*

ATF7IP was previously described to function not only as transcriptional repressor but also as transcriptional activator, as in addition to the repressive chromatin factors SETDB1 and MBD1, it was also found to interact and function with general transcription factors, RNA polymerase II, and the transcriptional activator SP1 [42,43]. Here, we considered the possibility that ATF7IP could directly control *Xist* expression, particularly because the effect of *Atf7ip* depletion alone (as described in Figure 1) mirrors that of *Xist* loss: *Xist* deletion alone only induces very minimal Xi-reactivation, but significantly enhances Xi-reactivation caused by DNA demethylation [20]. Therefore, it could be possible that ATF7IP functions as an activator of *Xist* in the context of XCI. In this scenario, depletion of *Atf7ip* could reduce *Xist* expression and coating of the chromosome by the RNA, which in turn could enhance Xi-reactivation in the presence of DNA methylation inhibitors.

**Figure 3:**
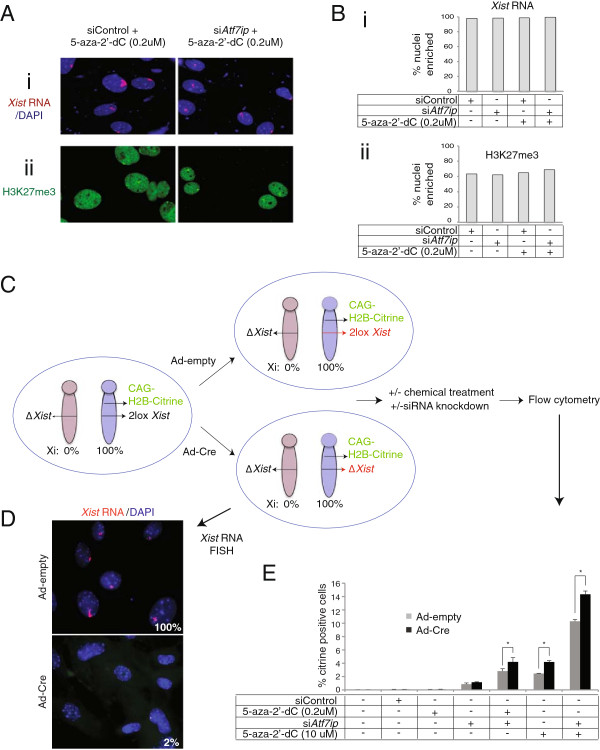
**Relationship between *Atf7ip *and *Xist. *(A)** (i) Representative FISH image for *Xist* RNA (red) in female DAPI-stained MEFs after 72 h of treatment with 5-aza-2’-dC (0.2 uM) and siRNA-mediated *Atf7ip* knockdown or GFP (control) knockdown. (ii) As in (i), but displaying an immunostaining image for H3K27me3 under the same conditions. **(B)** (i) The graph depicts the percentage of DAPI-stained nuclei (n = 200 per sample) with Xi-accumulation of *Xist* RNA for the conditions shown in **(Ai)** and additional control treatments. (ii) As in **(Bi)**, but for the Xi-accumulation of H3K27me3. **(C)** Diagram of the experimental approach to test the effect of combined *Xist* and *Atf7ip* depletion on Xi reactivation. Primary female MEFs with an Xi bearing a conditional loxP-flanked allele of *Xist* (2lox *Xist*) and a CAG-driven H2B-Citrine transgene in the *Hprt* locus, and an Xa without a functional *Xist* gene were obtained by crossing mice with the respective alleles. Cells were treated with either Adenovirus(Ad)-Cre or Ad-empty (empty adenoviral vector) for 24 h, then subjected to *Atf7ip* or control knockdowns and 5-aza-2’-dC treatments at various concentrations for an additional 72 h, and analyzed by flow cytometry. **(D)** Representative images of *Xist* RNA FISH in Ad-Null and Ad-Cre treated MEFs at the time of flow cytometry. The percentage of cells with Xi-like *Xist* RNA accumulations is given (200 cells counted). **(E)** Graph representing the percentage of H2B-citrine positive cells after 72 h of the indicated chemical and knockdown treatments. Grey bars indicate the results for Ad-empty treated (*Xist*-postive) cells and black bars indicate those for Ad-Cre treated (*Xist*-negative) cells. As siControl we used siRNAs with a scrambled sequence in this experiment.

We explored this idea by testing whether *Atf7ip* knockdown alters the Xi localization of *Xist* RNA, and the enrichment of the histone H3K27me3 and the chromatin regulator ASH2L, both of which are known to be recruited to the Xi in an *Xist* RNA-dependent manner [44,45]. Our FISH and immunostaining approaches demonstrated that *Atf7ip* knockdown, with or without a low dose 5-aza-2’-dC (0.2 uM), did not change the extent of Xi enrichment/localization of *Xist* RNA, H3K27me3, and Ash2l (Figure 3A, B, Additional file 6: Figure S6A, B), indicating that Atf7ip does not influence XCI by regulating *Xist* expression and the coating of the chromosome by *Xist* RNA, or by affecting the H3K27me3 pathway. We also compared the extent *Xist* RNA coating in cells that have undergone reactivation of the Xi-linked GFP reporter versus cells that remained GFP-negative upon treatment, and did not note significant differences in *Xist* RNA coating (Additional file 6: Figure S6C, D). The retention of the *Xist* RNA cloud in cells with Xi-reactivation supports the idea that *Xist* RNA coating is not lost as Xi-reactivation occurs.

Since these data suggested that *Atf7ip* does not control *Xist* expression or coating, we next determined the consequences of combined knockdown of *Atf7ip* and deletion of *Xist* in MEFs on the Xi-linked H2B-citrine reporter. We hypothesized that if ATF7IP is acting in the capacity of a transcriptional repressor directly on Xi linked genes, we would see an increase in the number of cells with Xi-reactivation when both *Atf7ip* and *Xist* are inhibited compared to the deletion of only one of these factors. To measure the relative contributions of *Xist* and *Atf7ip* to Xi-maintenance, we employed female MEFs carrying the histone H2B-Citrine reporter in the *Hprt* locus in *cis* to a conditional allele of the *Xist* gene on the Xi and assayed reactivation by FACS (Figure 3C). *Xist* was deleted in half of these MEFs by infection with a Cre-recombinase-encoding adenovirus (Ad-Cre), while the other half was treated with control adenovirus (Ad-Null) (Figure 3C). Efficient loss of *Xist* coating in almost all of the Cre-treated cells was confirmed by FISH (Figure 3D). MEFs positive and negative for *Xist*, respectively, were then subjected to *Atf7ip* knockdown and 5-aza-2’-dC treatment for 72 h to assess the contribution of *Xist* to Xi silencing under these conditions (Figure 3C, E).

Consistent with a previous report, *Xist* deletion led to a roughly two-fold increase in the number of cells displaying Xi-reactivation in MEFs treated with 5-aza-2’-dC at a high concentration (10 uM) (Figure 3E) [20], confirming the role of *Xist* in Xi-maintenance. Reporter activation was also enhanced by *Xist* deletion when MEFs were depleted for *Atf7ip*, both with and without 5-aza-2’-dC at low concentration (0.2 um) (Figure 3E). Furthermore, with approximately 14% cells being positive for histone H2B-citrine expression at 72 h of treatment, maximal reactivation of the H2B-citrine reporter was obtained when 10 uM (high) 5-aza-2’-dC, si*Atf7ip*, and loss of *Xist* were combined (Figure 3E). These findings further validate ATF7IP as a critical regulator of the Xi in somatic cells. Importantly, these observations argue against a role for ATF7IP in transcriptional activation of *Xist* and strongly point to an independent role in the repression of Xi-linked genes.

### ATF7IP’s repressive binding partners MBD1 and SETDB1 function in XCI

**Figure 4:**
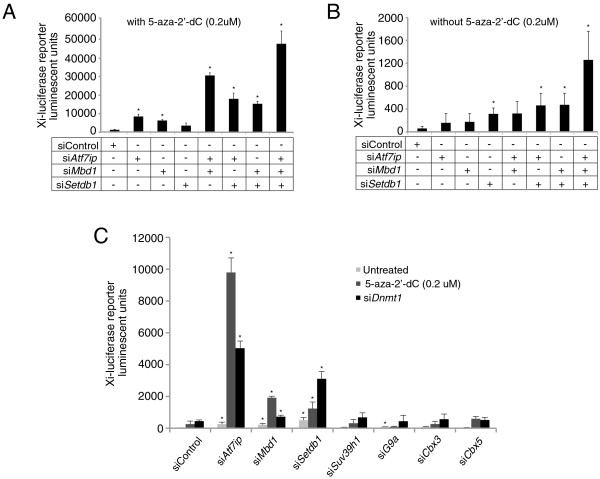
**Knockdown of factors in the repressive *Atf7ip *module that link the DNA methylation and H3K9methylation pathways also reactivates Xi-reporters. (A)** Female Xi-luciferase reporter MEFs (Xi^CAG-Luciferase^Xa^ΔXist^ MEFs) were treated indicated siRNAs and combinations thereof in the presence of 0.2 uM 5-aza-2’-dC, and luciferase activity was measured 72 h later. The Graph depicts the measured raw ALU values and error bars indicate the standard deviation of values from three individual wells with the same treatment in one experiment. * = *P* <0.05 relative to siControl (siGFP) by Student’s T-test. **(B)** As in **(A)**, except that the 5-aza-2’-dC treatment was omitted. **(C)** As in **(A)**, but Xi-luciferase reporter MEFs were treated with siRNAs targeting various regulators of H3K9methylation, without concomitant inhibition of DNA methylation (untreated), in the presence of 0.2 uM 5-aza-2’-dC, or with depletion of *Dnmt1* (si*Dnmt1*), and luciferase activity was measured 72 h later.

In its silencing context, ATF7IP acts as a bridging factor by binding the methyl-CpG DNA Binding Domain Protein 1 (MBD1) and the histone H3K9 trimethylase SETDB1, thereby coupling DNA methylation to histone H3K9 trimethylation and facilitating the conversion of di-methyl to tri-methyl H3K9 by SETDB1 and transcriptional repression [29-31,46]. Therefore, we hypothesized that ATF7IP could function together with MBD1 and SETDB1 on the Xi, and next addressed whether the factors implicated in ATF7IP’s autosomal gene silencing function also play a role in XCI by testing if depletion of *Mbd1* and *Setdb1* would similarly lead to Xi reactivation. Specifically, we assayed the effect of *Setdb1* and *Mbd1* knockdown on Xi-reporter activity both in the presence and absence of low concentration of 5-aza-2’-dC (0.2 uM), as we had previously observed that the effect of *ATF7IP* depletion was strongly enhanced by concomitant impairment of DNA methylation (Figure 1). Indeed, similar to *Atf7ip* depletion, knockdown of *Mbd1* or *Setdb1* activated the Xi-linked luciferase reporter more efficiently than control knockdown, both with and without low concentration of 5-aza-2’-dC (Figure 4A, B, Additional file 7: Figure S7A). Importantly, as seen before for *Atf7ip*, low levels of 5-aza-2’-dC dramatically increased the reactivation of the Xi-linked luciferase reporter due to knockdown of *Mbd1* or *Setdb1* (compare Figure 4A with Figure 4B, note the different Y-scales). We also found that the simultaneous depletion of any two of these factors induced a further increase in luciferase expression from the Xi both with and without 5-aza-2’-dC, and that the depletion of all three (*Atf7ip*, *Mbd1*, and *Setdb1*) had the most dramatic effect (Figure 4A, B). The additive enhancement of reactivation by combinatorial knockdown is likely due to hypomorphic effect of individual siRNA knockdowns that, when superimposed, reduce the overall silencing contribution of the *Mbd1-Atf7ip-Setdb1* arm in the XCI pathway, or may indicate additional functions of these proteins outside of the DNA/H3K9 methylation axis. Nevertheless, these data suggest that *Atf7ip* acts in the maintenance of X-inactivation within the *Mbd1-Atf7ip-Setdb1* pathway.

To further investigate whether the *Mbd1-Atf7ip-Setdb1* pathway is a specific gene silencing mechanism that is involved in the maintenance of XCI, and to examine whether other related silencing factors also have a role in XCI, we screened a selected panel of siRNAs validated to target other mediators of transcriptional silencing including heterochromatin proteins (*Cbx3* and *Cbx5*) and other H3K9 methyltransferases (*Ehmt2* (G9a) and *Suv39h1*) (Figure 4C) [47]. Despite efficient knockdown (Additional file 7: Figure S7B), our data do not implicate any of these additional factors in the maintenance of XCI even when tested with 5-aza-2’-dC or si*Dnmt1* sensitization (Figure 4C). This finding supports the conclusion that the three factors *Mbd1*, *Atf7ip*, and *Setdb1* play a specific role in XCI.

Taken together, our results suggest that DNA methylation, the *Mbd1-Atf7ip-Setdb1* pathway, and *Xist* RNA cooperate in maintaining the silent state of Xi-linked genes in differentiated female cells.

## Conclusions

In summary, we show that depletion of *Atf7ip* or of its known biochemical interactors *Mbd1* and *Setdb1* leads to Xi-reactivation. We also assayed the interaction of *Atf7ip* with two of the known major mediators of Xi-maintenance, *Xist* expression and DNA methylation. Our data reveal a strongly enhanced reactivation of the Xi when all these mechanisms are inhibited and implicate the *Mbd1-Atf7ip-Setdb1* pathway, described to couple DNA methylation and histone 3 lysine 9 trimethylation, in the maintenance of XCI in differentiated cells.

It remains unclear from our data whether ATF7IP is necessary for XCI *in vivo*, as, to our knowledge, no knockout mouse model has been described yet. *Atf7ip*’s pattern of expression is consistent with a role in XCI maintenance as *in situ* hybridization shows that it is ubiquitously expressed in d9.5 embryos with more specific tissue distribution at later time points [46]. Notably, ATF7IP’s repressive partners MBD1 and SETDB1 have very different knockout phenotypes, namely mild spatial learning defects and autoimmune disease in adult mice lacking *Mbd1*, and early embryonic lethality at 3.5 to 5.5 days post conception for the *Setdb1* deletion [48-50]. Given these different knockout phenotypes, it would be interesting to see whether *Atf7ip* tethers the two proteins, one binding methylated DNA and one mediating histone H3K9 trimethylation, only in specific developmental contexts, such as XCI. Notably, the effect of *Atf7ip*, *Setdb1*, or *Mbd1* depletion on the Xi was more clearly unmasked when other repressive mechanisms were also inhibited, suggesting that the knockout animals may not reveal problems with XCI due to redundancy of silencing mechanisms acting on the Xi.

The identification of *Atf7ip* as a factor involved in XCI maintenance suggests that the downstream effect of its binding partner *Setdb1*, namely enzymatic conversion of H3K9me2 to H3K9me3, may also be required on the Xi. H3K9 methylation has previously been reported to play a role in XCI on the basis of enrichment on the Xi detected with a pan-methyl H3K9 antibody in mouse and human cells, though it is unclear if the antibody used in these studies had cross-reactivity to H3K27me3 [23-25]. Other studies comparing H3K27me3 and H3K9me2 by ChIP and by immunostaining established that both marks are increased on the Xi in MEFs relative to the active X [26,27] (Figure 2). In human cells, the Xi has an H3K9me3 domain distinct from the *Xist* and H3K27me3-enriching territory of the Xi [51]. Interaction of these domains is proposed to occur through the HBiX protein bridging HP1, a protein binding HK9me3, and SMCHD1, a protein required for methylation of CpG islands on the Xi and recruited by *Xist* to the H3K27me3 domains [52]. This bridging of H3K27me3 and H3K9me3 domains is thought to be necessary for Xi compaction, however, depletion of HBiX or SMCHD1 by siRNA did not lead to upregulation of Xi-linked genes [52], leaving an open question as to whether H3K9me3 has silencing function in XCI and whether that function is dependent on HP1 and HBiX binding.

It remains unclear whether knockdown of *Setdb1*, though a known histone H3K9 trimethyltransferase, would deplete H3K9me3 on the Xi, since the *Setdb1* knockout has not been shown to lead to global alterations in H3K9me3 levels nor DNA methylation in early embryonic mouse cells when assayed by immunostaining and bisulfite sequencing of IAP elements, respectively [48]. In addition, the H3K9 methyl reader heterochromatin proteins Cbx1, Cbx3, and/or Cbx5 are thought to mediate transcriptional repression downstream to H3K9 methylation, yet, in our study, knockdown of *Cbx3* or *Cbx5* individually had no effect on the reactivation of the Xi in somatic cells (Figure 4C). Furthermore, we did not see strong Xi enrichment of *Atf7ip* or H3K9me3 by immunostaining (Figure 2, Additional file 8: Figure S8). However, we observed that in a fraction of cells a subdomain of the H3K27me3-demarcated Xi co-localizes with a punctum of H3K9me3 enrichment (Additional file 8: Figure S8). ChIP-seq studies of H3K9me3 and the analysis of DNA methylation patterns with and without knockdown of *Atf7ip*, *Setdb1*, and *Mbd1* will be necessary to refine the role of the *Atf7ip/Setdb1/Mbd1* pathway on the Xi and to determine whether genes on the Xi have different propensities for regulation through this pathway. To explain the combinatorial effect of DNA methylation with interference of the *Mbd1-Atf7ip-Setdb1* pathway, we hypothesize that some DNA methylation loss is necessary to ‘unlock’ the stably silenced chromatin. However, the relationship between H3K9me3 and DNA methylation may be reciprocal and H3K9me3 could also recruit the DNA methylation machinery [53].

Finally, ATF7IP has also been shown to interact with CDYL, a protein implicated in the maintenance of H3K9me2 on the Xi, which requires H3K27me3 and H3K9me2 for its Xi accumulation on the Xi [27]. Therefore, Atf7ip may contribute to an extensive regulatory network that links various repressive chromatin pathways to contribute to the maintenance of the Xi.

## Methods

### Cell culture methods

Mouse ESCs were grown on irradiated DR4 MEFs in standard media (DMEM supplemented with 15% FBS, nonessential amino acids, L-glutamine, penicillin-streptomycin, β-mercaptoethanol, and 1000 U/mL LIF). For induction of differentiation, cells were feeder-depleted for 45 min and subsequently plated at a density of 5.0 × 10^4^ cells/ 6-well in MEF media (same as ESC media except that 10% FBS were used and LIF was omitted). One day later, MEF media was supplemented with 1 μM all-trans retinoic acid (Sigma) and refreshed every 2 days, for a total of 5 days. Male ESCs carrying the tet-inducible promoter in front of the endogenous *Xist* allele were induced to differentiate with 1 μg/mL doxycycline in standard ESC media for 21 h [41]. MEFs were cultured in MEF media and derived at embryonic day 14.5 from timed matings of mice with the appropriate genotypes (see below).

### Generation of X-linked reporter MEFs

*Hprt*-CAG-Luciferase and *Hprt*-CAG-H2B-citrine reporters were engineered in male V6.5 ESCs. These ESCs were modified by two-step targeting as described in Additional file 2: Figure S2, closely following a strategy originally described for the *Col1A* locus in [33]. First, an FRT-flanked neomycin resistance cassette and a hygromycin resistance gene that has an FRT site embedded in its 5’coding region but lacks a promoter and an ATG initiation codon, were placed into *Hprt* locus by homologous recombination, deleting 1,313 bp of *Hprt* sequence between homology arms including the third exon of *Hprt*. To place the ‘FRT-neo-FRT-hygro’ cassette into the *Hprt* locus, the *ColA1* homology arms of the *Col1A*-FRT-hygro-pA ‘homing’ vector described in [33] were replaced with homology regions targeting the *Hprt* locus. The *Hprt*-FRT-hygro-pA ‘homing’ vector was introduced into V6.5 ESCs by electroporation followed by selection with 350 ug/mL G418. DNA from picked clones was analyzed for proper targeting by southern blotting using a BglII digest as outlined in Additional file 2: Figure S2. Second, using FLPe-mediated recombination, the CAG-luciferase or CAG-H2B-citrine transgene was introduced into one ESC clone carrying the *Hprt-*FRT hygro-pA ‘homing’ site, specifically into the FRT site in front of the hygromycin open reading frame, using the plasmids pBS32-CAG-luciferase or pBS32-CAG-H2B-citrine as shown in Additional file 2: Figure S2. Co-electroporation of these plasmids with a plasmid expressing Flpe recombinase was followed by hygromycin treatment selecting for ESC clones that have the pgk promoter driving the hygromycin resistance gene due to integration of the entire pBS32 plasmid sequence. DNA from selected clones was digested with BglII and screened by southern blotting using 3’ external probe for correct FLP recombination (Additional file 2: Figure S2, data not shown). The pBS32 vector was made by exchanging the tetracycline-responsive operating binding sequence in the pgkATGfrt vector described in [33] with the constitutive CAG promoter. Luciferase was introduced into pBS32 by Gateway® cloning (Life Technologies). To make pBS32 vector compatible for Gateway cloning (pBS32-GW), a Gateway cassette with attR sites and a ccdB gene was flanked by SgrAI restriction sites and ligated into a unique EcoRI site on pBS32. One-step BP and LR cloning was performed with pDonr221 entry vector, attB-primer amplified firefly luciferase from pGL3 vector (Promega) and pBS32-GW. The pBS32-CAG-H2B-citrine was constructed analogously using a H2B-citrine template kindly provided by Michael Elowitz (Caltech). Luciferase and H2B-citrine reporter ESCs were microinjected by the UCLA transgenic core into C57BL/6 blastocysts to produce chimeric mice following standard procedures. High agouti coat color male chimeras were bred with C57BL/6 females for germline transmission. For skewing of XCI to the luciferase-bearing X chromosome, X-linked reporter male mice were bred with female mice heterozygous for an *Xist* knockout (Δ*Xist*) allele [34]. For the experiments requiring simultaneous deletion of *Xist* and reporter quantification, 2lox *Xist*[54] and *Hprt*-H2B-Citrine alleles were bred until a spontaneous recombination event brought the alleles *in cis*. The recombinant males were subsequently bred to Δ*Xist* females to skew X inactivation to the reporter-bearing X chromosome in the embryos. All animal experiments were in accordance with the legislation of the UCLA Animal Research Committee.

### Knockdown, overexpression, and chemical treatments of MEFs

For Xi-reactivation assays, MEFs at passage 1 or 2 post-derivation were seeded at a density of 60,000 cells per 12-well well and chemicals in MEF media were added and incubated for 72 h before further analysis (by FISH, immunostaining, or reporter assays). 5-aza-2’-dC (Sigma A3656) was dissolved in DMSO and added at indicated concentrations. Across all conditions in a given experiment, final DMSO concentration (always below 0.1%) and final volumes of media were kept constant across samples. Knockdown with siRNA was performed by reverse transfection at 25 nM final concentration of siRNA. Briefly, a cell suspension was added to a pre-incubated mixture of Lipofectamine RNAimax, 100 uL of reduced serum Opti-MEM media, and siRNA. siRNAs used in this study included *Atf7ip* (Ambion, AM16706), *Dnmt1* (Ambion, AM161526), *Mbd1* (Dharmacon, MU-056829-01), *Setdb1* (Dharmacon, D-040815-04), *Suv39h1* (Dharmacon MU-046141-10), *Ehmt2* (Dharmacon, MU-053728-03), *Cbx3* (Dharmacon MU-044218-02), *Cbx5* (Dharmacon MU-040799-02), Luciferase (Dharmacon, D-001210-02), Scramble (Ambion, AM4636), and GFP (Dharmacon, P-002048-01). For experiments involving multiple knockdowns, control siRNA (against either Scramble, GFP, or Luciferase depending upon experiment as indicated in figure legends) was added to equalize the final siRNA concentration across all conditions. For overexpression of Flag-tagged *Atf7ip*, the *Atf7ip* cDNA was introduced into the pMX retroviral vector by In-Fusion® cloning (Clontech) of the *Atf7ip* cDNA and incorporation of the FLAG tag by PCR at the N-terminal end. The pMX retrovirus was generated in transfected platE cells, and MEFs were transduced as previously described [7].

### Luciferase assay

Chemical and/or siRNA treatments of MEFs were performed in triplicate 12-well wells for 72 h and cells were lysed with 200 uL passive lysis buffer (PLB, Promega) for 20 min at room temperature on an orbital shaker. Lysates were cleared by 30 s of centrifugation at 12,000 × *g* and 20 uL were assayed for luciferase activity with 50 uL of LARI reagent (Promega) on a GloMax microplate luminometer (Promega).

### RT-qPCR analysis

Cells were harvested from a 6-well format in Trizol (Invitrogen) and RNA purification was performed with the RNeasy kit (Qiagen) according to manufacturer’s instructions with on-column DNAse treatment (Qiagen). cDNA was prepared using SuperScript III (Invitrogen) with random hexamers and RT-qPCR was performed using a Stratagene Mx3000 thermocycler with primers listed in Additional file 9: Table S1. Results were normalized to Gapdh by the ΔCt method.

### Western blot analysis

Xi-luciferase MEFs were treated as described above and one well was used to confirm luciferase reactivation. Cells in the other well were lysed in RIPA buffer and sonicated (30 s on, 30 s off, cycled for 5 min). Equal volume from each sonicated lysate was loaded onto a 4% to 12% 10-well 1.5 mm Bis-Tris gel (NuPAGE). Proteins were transferred onto a nitrocellulose membrane overnight at 10 V in 4°C. Membranes were incubated overnight at 4°C with 1:2,500 dilution of polyclonal rabbit-anti-Atf7ip antibody (Nakao lab). Monoclonal mouse anti-alpha-Tubulin (Calbiochem CP06) was used as loading control at 1:1,000 dilution overnight at 4°C. Quantification was performed on the Odyssey scanner (Li-Cor) with Image Studio software (Li-Cor) and band intensity was normalized to alpha-Tubulin loading control.

### Immunofluorescence, FISH, and chromosome paint analysis

Cells were plated on glass coverslips, washed once with PBS, and fixed for 10 min in 4% paraformaldehyde [7]. Immunostaining with antibodies against ATF7IP (Abcam 84497), H3K27me3 (Active Motif 39155), H3K9me2 (Cosmo Bio MCA-MABI0007-100-EX), H3K9me3 (Active Motif 39161, AbCam ab8898), Ash2L (Bethyl a300-107a), FLAG M2 (Sigma F3165), GFP (Nacalai USA Inc., 04404–84), and FISH with DNA probes against *Xist* and *Atrx* and IF/FISH combinations thereof were performed as previously reported and mounted with Prolong Gold reagent with DAPI [38]. BAC templates for *Atrx* FISH probes were obtained from the BACPAC resource (*Atrx* RP23-265D6).

### Flow cytometry

Cells were trypsinized, washed in PBS, loaded through cell strainer caps (BD Biosciences) and analyzed on a FACSDiva machine (BD Biosciences) with FlowJo software (Tree Star, Inc.).

## Competing interests

The authors declare that they have no competing interests.

## Authors’ contributions

AM participated in planning of the project, experimental data generation, data interpretation, manuscript writing, and provided some financial support. AS participated in planning of the project, experimental data generation, data interpretation, and editing of manuscript. ERG participated in experimental data generation. GB performed bioinformatics analysis. SP established various FISH/immunostaining procedures. KP conceived of the study and supervised the project, as well as participated in data interpretation, manuscript writing, and provided financial support. KP had final approval of the manuscript. All authors read and approved the final manuscript.

## Supplementary Material

Additional file 1: Figure S1 Validation of the *Atf7ip* knockdown approach. This figure includes western blot and immunostaining images to support knockdown of ATF7IP protein and transcript levels. **(A)** Western blot of cells treated as in Figure 1A, except in the presence of 5-aza-2’-dC (0.2 uM) with an antibody against ATF7IP (band size approximately 220 kDa), with alpha-tubulin loading control. **(B)** Quantification of band intensity from the western blot shown in **(A)** normalized to alpha-tubulin loading control. **(C)** Female MEFs were treated with siRNAs targeting *Atf7ip* and Luciferase (siControl), and *Atf7ip* transcript levels were determined by RT-qPCR*.* The data were normalized to the control treatment and to *Gapdh* expression. Error bars indicate one standard deviation from three independent experiments. **(D)** Representative immunostaining image for ATF7IP (green) and FLAG (red) on female MEFs infected with a retrovirus encoding FLAG-tagged *Atf7ip* (pMX-*Atf7ip*)*,* 72 h after infection. DAPI marks the nuclei. Note, in the image only the nucleus on the left expresses FLAG-*Atf7ip* and is therefore detected with the FLAG-antibody. **(E)** MEFs were infected with the pMX-*Atf7ip* retrovirus as indicated and, 72 h later, treated with siRNAs targeting *Atf7ip* and siControl (targeting luciferase or GFP), respectively, for another 72 h. Subsequently, *Atf7ip* transcript levels were determined by RT-qPCR*.* The data were normalized to the siControl treatment and to *Gapdh* expression. Error bars indicate one standard deviation from three independent experiments. * = *P* <0.01 by Student’s T-test. Click here for file

Additional file 2: Figure S2 Construction of the X-linked luciferase reporter. This figure includes a diagram of targeting strategy to generate X-linked reporter transgenic mice. Targeting strategy for the generation of X-linked reporter ESCs. Top: Schematic representation of the *Hprt* locus on the X chromosome, with exons shown as gray boxes, and locations of the homology arms used to recombine the FRT-Hygro-pA ‘homing cassette’ in mouse ESCs. Additionally, the location of the 3’ probe and BglII restriction enzyme digest strategy used in Southern bloting to confirm targeting, are indicated. Note that this targeting strategy deletes exon 3 of *Hprt*. Middle: The luciferase cDNA was cloned into the pBS32 vector. Bottom: Co-electroporation of the pBS32-CAG-Luciferase vector and a FLPe expression vector into FRT-Hygro-pA ‘homing cassette’ - bearing mouse ESCs, and subsequent hygromycin selection ensures the survival of ESC clones with recombination of the FRT sites, leading to loss of the PGKneopA cassette and insertion of luciferase gene (as described in [33]). Click here for file

Additional file 3: Figure S3 The X-luciferase reporter is subject to XCI and sensitive to loss of DNA methylation. This figure includes a luciferase assay from the resulting transgenic MEFs to demonstrate that the X-linked luciferase reporter is silenced when located on the Xi and reactivates in response to interference with DNA methylation. **(A)** Top: Schematic of the X chromosomes in female MEFs carrying (i) the heterozygous luciferase reporter transgene (as described in **(A)**) on the Xi in 100% of the cells (Xi^CAG-Luciferase^Xa^ΔXist^ MEFs), and (ii) in female MEFs heterozygous for the luciferase reporter transgene without interference of *Xist* function, so that the luciferase reporter has a 50% chance of being silenced as part of the Xi. Bottom: Graph summarizing the luciferase values for MEFs displayed above (separated by dashed line) treated with 5-aza-2’-dC at the indicated concentrations (or DMSO vehicle) for 72 h. Error bars indicate standard deviation of raw ALU values from three individual wells with the same treatment condition from one representative experiment. Note, luciferase values are close to background when the reporter is on the Xi in all cells in the population (left, untreated), but increase when DNA methylation is impaired. In cells with random XCI (right, untreated), high luciferase signal can be detected in the untreated condition since approximately half the cells express the reporter from the Xa. **(B)** MEFs were treated with siRNAs targeting *Dnmt1* and siControl (siScramble) as described in Figure 1C, D, and *Dnmt1* transcript levels were determined by RT-qPCR*.* The data were normalized to the siControl treatment and to *Gapdh* expression. Error bars indicate one standard deviation from triplicate RT-qPCR measurements in one representative experiment. Click here for file

Additional file 4: Figure S4 DNA methlyation levels at the luciferase reporter gene in Xi^CAG-Luciferase^Xa^ΔXist^ MEFs. This figure summarizes reduced representation bisulfite sequencing data for the reporter locus before and after treatment with 5-aza-2’-dC. **(A)** IGV browser view of reduced representation bisulfite sequencing data within the approximately 3 kB reporter transgene (refer to Additional file 2: Figure S2) from two independent batches of Xi^CAG-Luciferase^Xa^ΔXist^ MEFs. The height of the blue bars represents % methylation at the individual CpG. Sites covered by RRBS are indicated. **(B)** Histograms of single CpG RRBS DNA methylation values across the luciferase reporter insert on the Xi in untreated Xi^CAG-Luciferase^Xa^ΔXist^ MEFs. The number of CpGs covered by RRBS is given. **(C)** As in **(B)**, except that the methylation values for Xi^CAG-Luciferase^Xa^ΔXist^ MEFs treated with 5-aza-2’-dC (10 uM) are shown, indicating a drop in overall methylation compared to **(B)**. Click here for file

Additional file 5: Figure S5 Effect of *Atf7ip* depletion on the expression of the Xi-GFP and Xi-linked H2B citrine reporter. This figure parallels Figure 1, and shows the actual fluorescent intensities of the Xi-linked fluorescent reporters upon various treatments. **(A)** Top: (Left) Schematic of the X chromosomes in female reporter MEFs heterozygous for the CAG-GFP reporter. Due to random XCI, the GFP is on the Xi in approximately half the population. (Right) When in conjunction with the *Xist* knockout allele, the CAG-GFP reporter is on the Xi in 100% of the cells (Xi^CAG-H2BCitrine^Xa^ΔXist^) due to skewing with *Xist* deletion. Bottom: GFP fluorescence was quantified by flow cytometry in the MEFs depicted above, treated with the indicated conditions for 72 h, and the FACS results are displayed. **(B)** (i) Schematic of the X chromosomes in female reporter MEFs carrying a heterozygous H2B-Citrine reporter (within the *Hprt* locus) on the Xi in all cells of the population (Xi^CAG-H2BCitrine^Xa^ΔXist^ MEFs). (ii) Citrine fluorescence was quantified by flow cytometry in these MEFs treated with the indicated conditions for 72 h. Note siControl knockdown produced significant background citrine fluorescence signal compared to the untreated sample. (iii, iv) FACS plots for data summarized in (ii) with initial cell gating (iii) and citrine-positive cell gating shown (iv). Click here for file

Additional file 6: Figure S6 *Atf7ip* does not control the *Xist*-dependent accumulation of the chromatin regulator ASH2L on the Xi. This figure supports Figure 2 with FISH and immunostaining to demonstrate that *Atf7ip* knockdown, with or without a low dose 5-aza-2’-dC (0.2 uM), does not change the extent of Xi enrichment/localization of Ash2l. It also shows that rates of *Xist* RNA coating of the Xi are not affected in cells that display the reactivation of the Xi-linked-GFP reporter. **(A)** Immunostaining for ASH2L (red) on MEFs 72 h after transfection of siRNAs targeting *Atf7ip* or control knockdown (targeting GFP), respectively, and treatment with 5-aza-2’-dC (0.2 uM) similar to the experiment described in Figure 2A. Representative images are shown and DAPI was used to demarcate the nucleus. **(B)** Graph summarizes the proportion of DAPI-stained nuclei (n = 200 per sample) with and Xi-like accumulation of ASH2L for the experiment described in (A) and additional conditions. **(C)** Xi^CAG-H2BCitrine^Xa^ΔXist^ MEFs were treated for 72 h with *Atf7ip* siRNA and 5-aza-2’-dC (0.2 um) or with 5-aza-2’-dC 10.0 uM alone. Given is a representative IF/FISH image for *Xist* (red) and GFP (green) that depicts a cell with GFP reactivation that contains an *Xist* RNA coated chromosome. **(D)** Quantification of *Xist* RNA coating in GFP + and GFP-negative cells from the experiment described in **(C)**, indicating the *Xist* RNA coating is maintained in cells displaying reactivation of the Xi-linked GFP. Click here for file

Additional file 7: Figure S7 Confirmation of knockdown of *Atf7ip*-related factors. This figure supports Figure 4 to show that extent of knockdown of various transcripts by RT-PCR in the same experiments. **(A)** MEFs were treated with siRNAs targeting the indicated genes or siControl (siGFP) as described in Figure 4 and respective transcript levels were determined by RT-qPCR (that is, 40% of *Atf7ip* transcription remaining after si*Aft7ip* treatment, 40% of *Mbd1* transcript remaining after si*Mbd1* treatment, and so on)*.* The data were normalized to the siGFP (control) treatment and to *Gapdh* expression. Error bars indicate one standard deviation from triplicate RT-qPCR measurements in one representative experiment. **(B)** As in **(A)**, but for the experiment shown in Figure 4C in the untreated condition (without 5-aza-2’-dC or si*Dnmt1*). Click here for file

Additional file 8: Figure S8 H3K9me3 localization relative to the H3K27me3 Xi-domain. This figure contains immunostaining images for H3K9me3 relative to H3K27me3 to identify the Xi. Representative immunostaining images of female MEFs for H3K9me3 (green, with two different antibodies termed Active Motif and AbCam) and H3K27me3 (red) with zoomed-in views of a single nucleus. DAPI was used to demarcate the nuclei. Click here for file

Additional file 9: Table S1 Primers for RT-PCR. Primer sequences used for RT-PCR analysis. Click here for file
