# Supplementary figures and images for: The Mbd1-Atf7ip-Setdb1 pathway contributes to the maintenance of X chromosome inactivation
Source: Epigenetics Chromatin. 2014 Jun 26;7:12. doi: 10.1186/1756-8935-7-12 (PMC4099106; doi:10.1186/1756-8935-7-12)

# Supplemental Figure 1

A

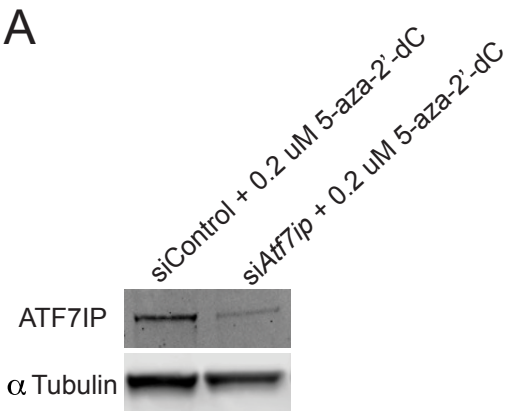

B

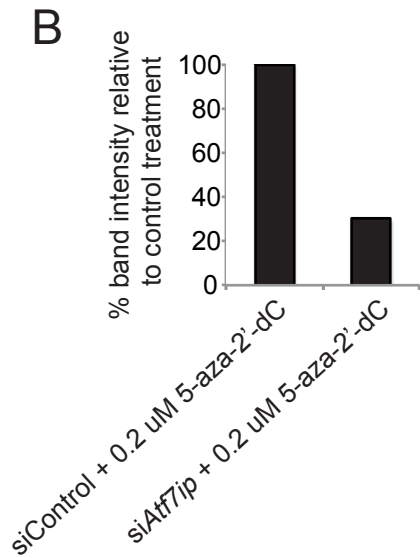

C

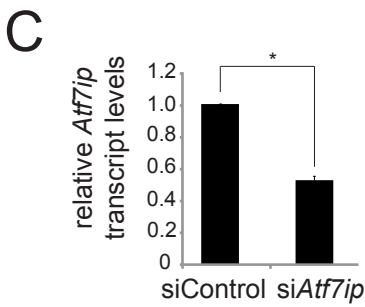

D

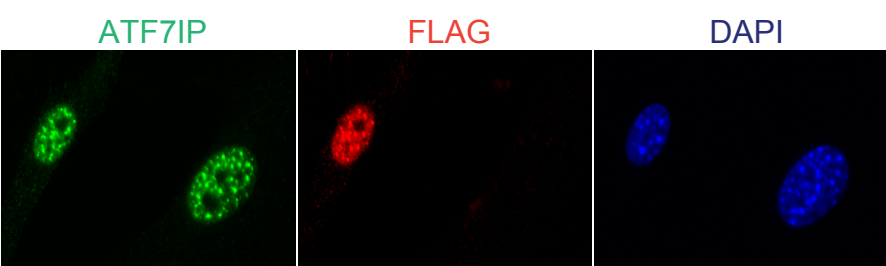

E

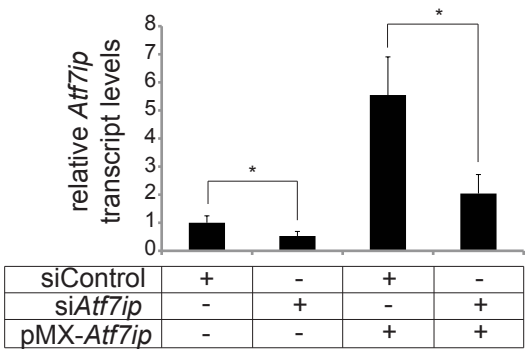

Supplement: Additional file 1: Figure S1 — Validation of the Atf7ip knockdown approach. This figure includes western blot and immunostaining images to support knockdown of ATF7IP protein and transcript levels. (A) Western blot of cells treated as in Figure 1A, except in the presence of 5-aza-2’-dC (0.2 uM) with an antibody against ATF7IP (band size approximately 220 kDa), with alpha-tubulin loading control. (B) Quantification of band intensity from the western blot shown in (A) normalized to alpha-tubulin loading control. (C) Female MEFs were treated with siRNAs targeting Atf7ip and Luciferase (siControl), and Atf7ip transcript levels were determined by RT-qPCR. The data were normalized to the control treatment and to Gapdh expression. Error bars indicate one standard deviation from three independent experiments. (D) Representative immunostaining image for ATF7IP (green) and FLAG (red) on female MEFs infected with a retrovirus encoding FLAG-tagged Atf7ip (pMX-Atf7ip), 72 h after infection. DAPI marks the nuclei. Note, in the image only the nucleus on the left expresses FLAG-Atf7ip and is therefore detected with the FLAG-antibody. (E) MEFs were infected with the pMX-Atf7ip retrovirus as indicated and, 72 h later, treated with siRNAs targeting Atf7ip and siControl (targeting luciferase or GFP), respectively, for another 72 h. Subsequently, Atf7ip transcript levels were determined by RT-qPCR. The data were normalized to the siControl treatment and to Gapdh expression. Error bars indicate one standard deviation from three independent experiments. * = P <0.01 by Student’s T-test. [file 1756-8935-7-12-S1.pdf]

# Supplemental Figure 2

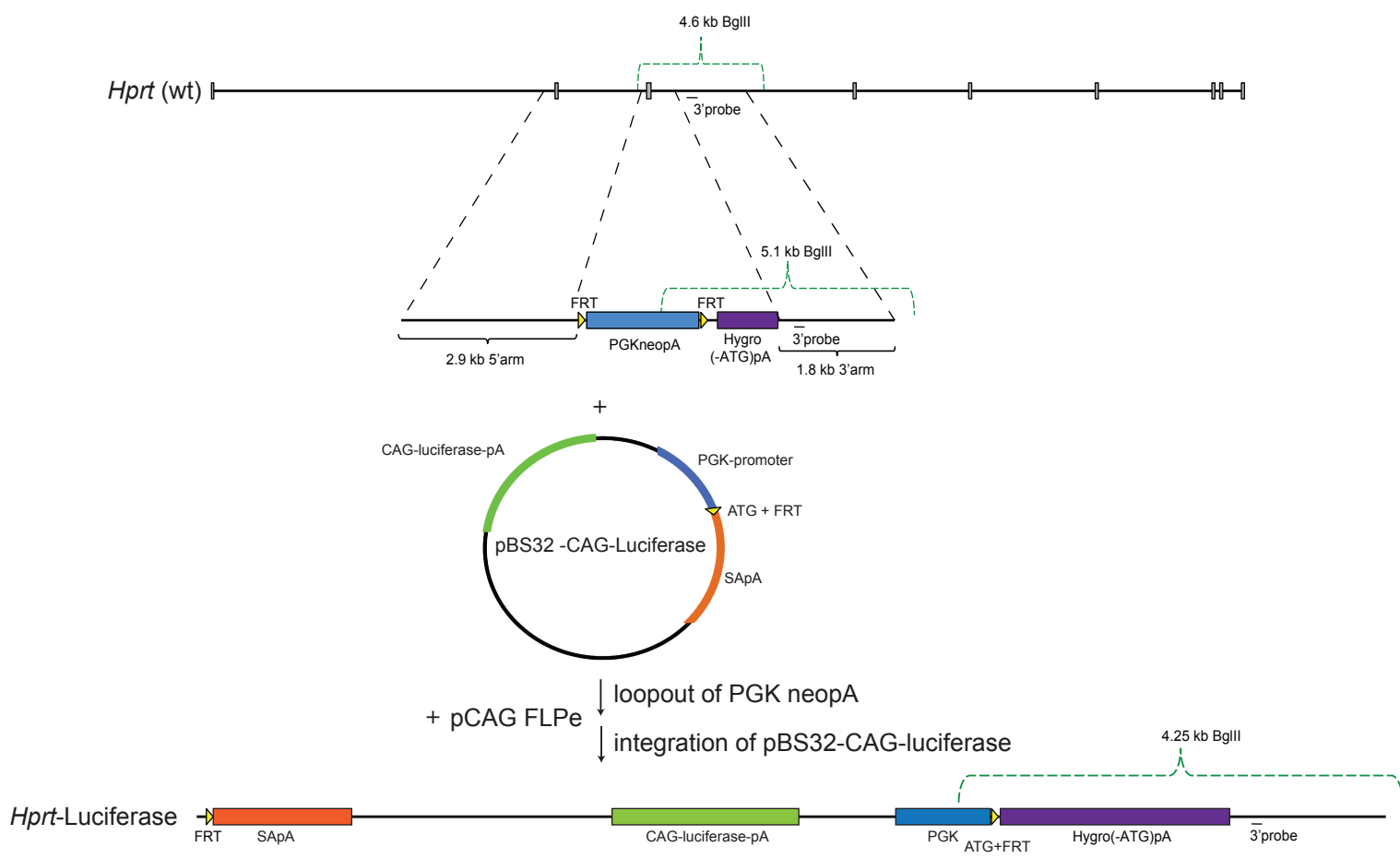

Supplement: Additional file 2: Figure S2 — Construction of the X-linked luciferase reporter. This figure includes a diagram of targeting strategy to generate X-linked reporter transgenic mice. Targeting strategy for the generation of X-linked reporter ESCs. Top: Schematic representation of the Hprt locus on the X chromosome, with exons shown as gray boxes, and locations of the homology arms used to recombine the FRT-Hygro-pA ‘homing cassette’ in mouse ESCs. Additionally, the location of the 3’ probe and BglII restriction enzyme digest strategy used in Southern bloting to confirm targeting, are indicated. Note that this targeting strategy deletes exon 3 of Hprt. Middle: The luciferase cDNA was cloned into the pBS32 vector. Bottom: Co-electroporation of the pBS32-CAG-Luciferase vector and a FLPe expression vector into FRT-Hygro-pA ‘homing cassette’ - bearing mouse ESCs, and subsequent hygromycin selection ensures the survival of ESC clones with recombination of the FRT sites, leading to loss of the PGKneopA cassette and insertion of luciferase gene (as described in [33]). [file 1756-8935-7-12-S2.pdf]

# Supplemental Figure 3

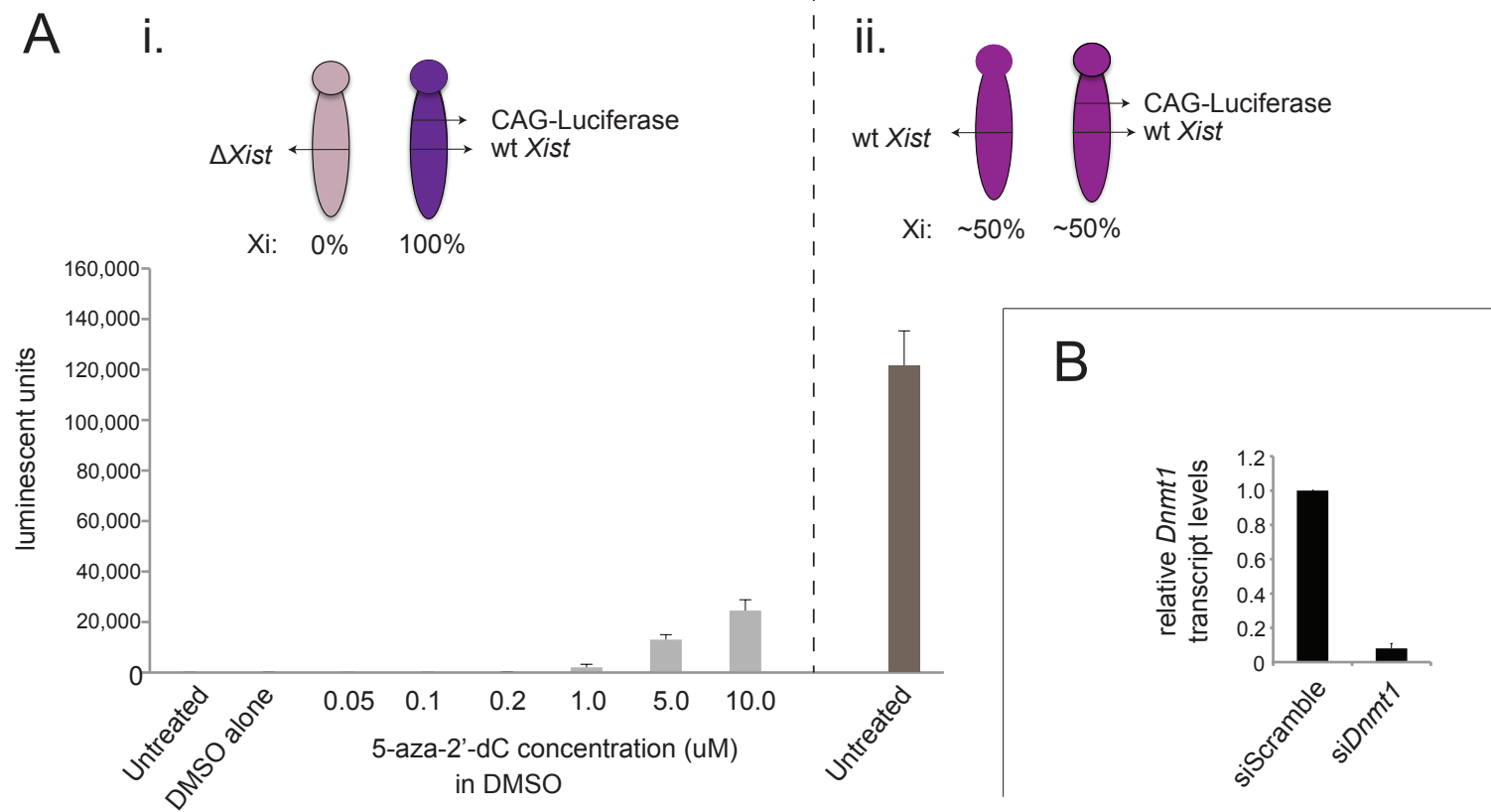

Supplement: Additional file 3: Figure S3 — The X-luciferase reporter is subject to XCI and sensitive to loss of DNA methylation. This figure includes a luciferase assay from the resulting transgenic MEFs to demonstrate that the X-linked luciferase reporter is silenced when located on the Xi and reactivates in response to interference with DNA methylation. (A) Top: Schematic of the X chromosomes in female MEFs carrying (i) the heterozygous luciferase reporter transgene (as described in (A)) on the Xi in 100% of the cells (XiCAG-LuciferaseXaΔXist MEFs), and (ii) in female MEFs heterozygous for the luciferase reporter transgene without interference of Xist function, so that the luciferase reporter has a 50% chance of being silenced as part of the Xi. Bottom: Graph summarizing the luciferase values for MEFs displayed above (separated by dashed line) treated with 5-aza-2’-dC at the indicated concentrations (or DMSO vehicle) for 72 h. Error bars indicate standard deviation of raw ALU values from three individual wells with the same treatment condition from one representative experiment. Note, luciferase values are close to background when the reporter is on the Xi in all cells in the population (left, untreated), but increase when DNA methylation is impaired. In cells with random XCI (right, untreated), high luciferase signal can be detected in the untreated condition since approximately half the cells express the reporter from the Xa. (B) MEFs were treated with siRNAs targeting Dnmt1 and siControl (siScramble) as described in Figure 1C, D, and Dnmt1 transcript levels were determined by RT-qPCR. The data were normalized to the siControl treatment and to Gapdh expression. Error bars indicate one standard deviation from triplicate RT-qPCR measurements in one representative experiment. [file 1756-8935-7-12-S3.pdf]

# Supplemental Figure 4

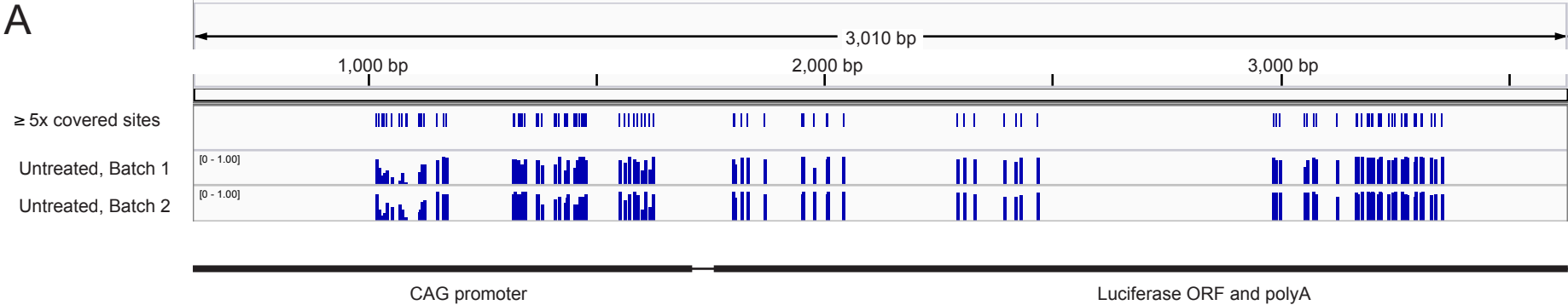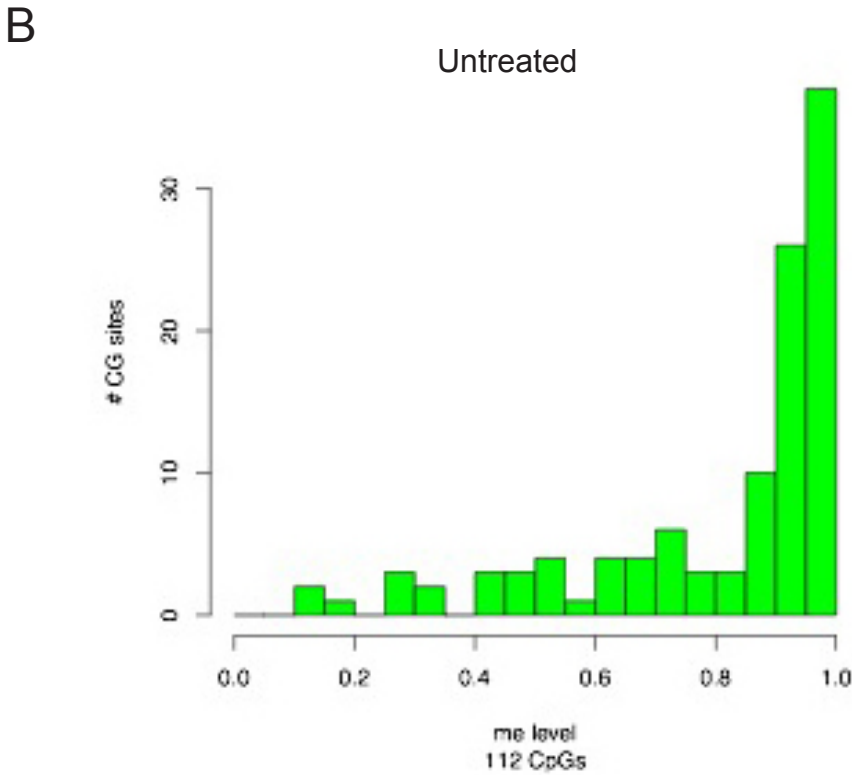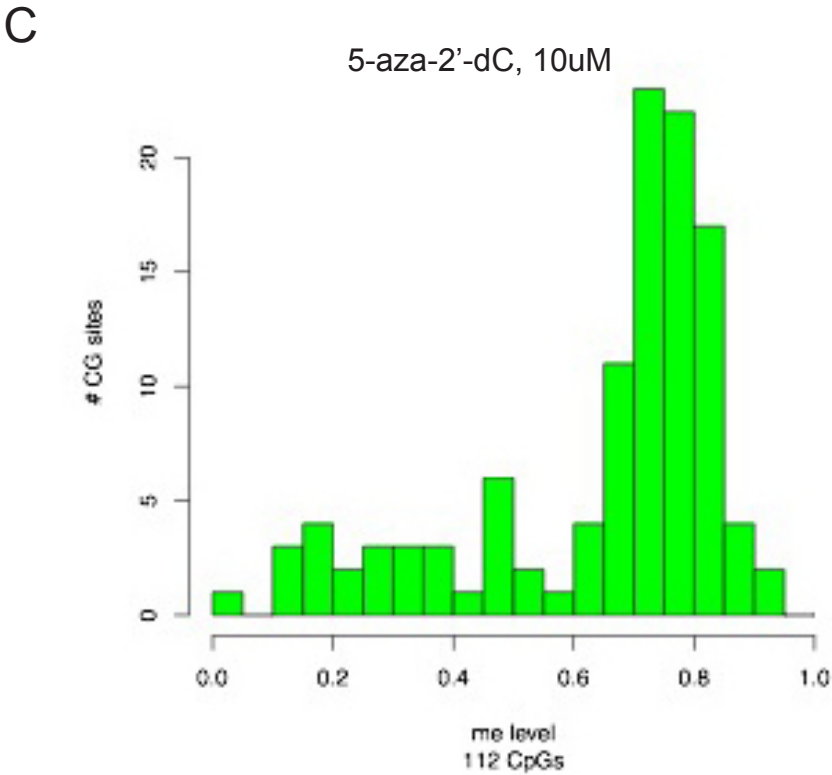

Supplement: Additional file 4: Figure S4 — DNA methlyation levels at the luciferase reporter gene in XiCAG-LuciferaseXaΔXist MEFs. This figure summarizes reduced representation bisulfite sequencing data for the reporter locus before and after treatment with 5-aza-2’-dC. (A) IGV browser view of reduced representation bisulfite sequencing data within the approximately 3 kB reporter transgene (refer to Additional file 2: Figure S2) from two independent batches of XiCAG-LuciferaseXaΔXist MEFs. The height of the blue bars represents % methylation at the individual CpG. Sites covered by RRBS are indicated. (B) Histograms of single CpG RRBS DNA methylation values across the luciferase reporter insert on the Xi in untreated XiCAG-LuciferaseXaΔXist MEFs. The number of CpGs covered by RRBS is given. (C) As in (B), except that the methylation values for XiCAG-LuciferaseXaΔXist MEFs treated with 5-aza-2’-dC (10 uM) are shown, indicating a drop in overall methylation compared to (B). [file 1756-8935-7-12-S4.pdf]

# Supplemental Figure 5

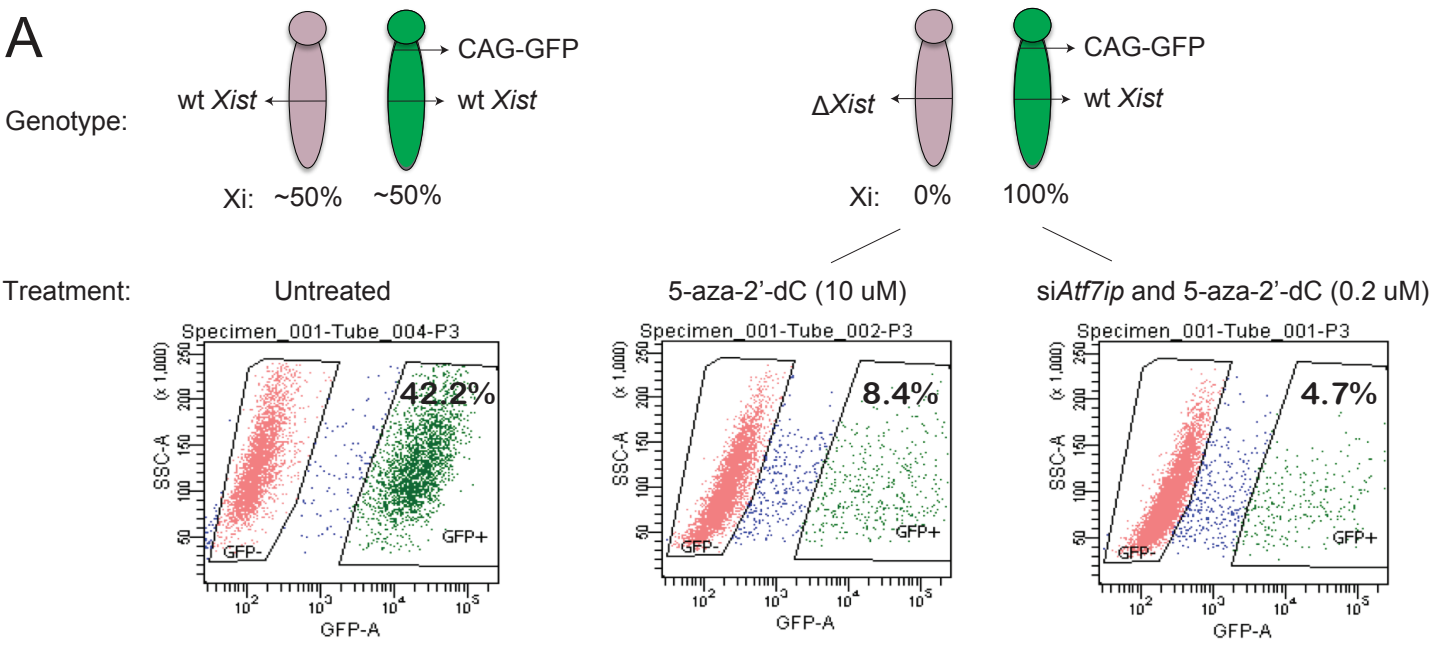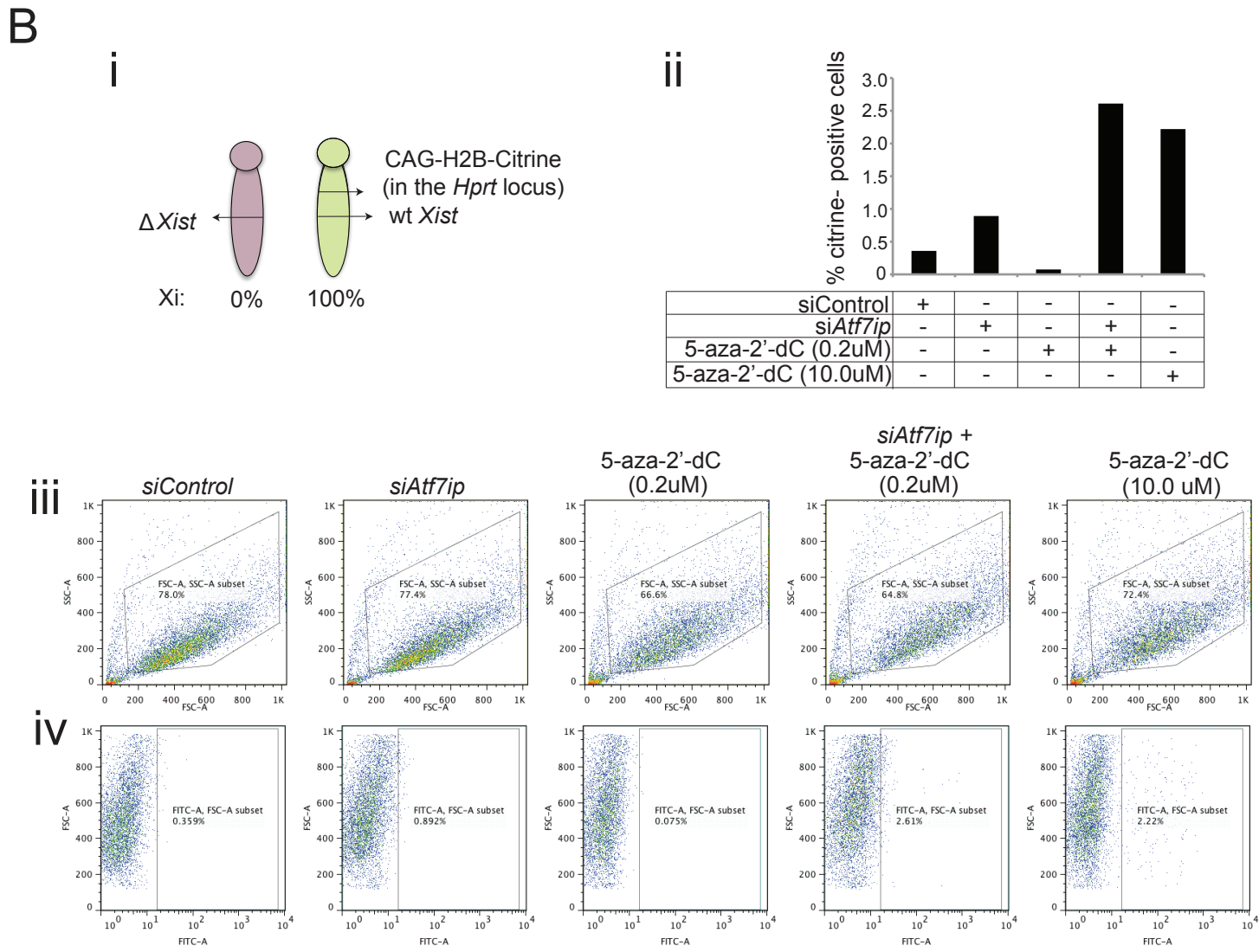

Supplement: Additional file 5: Figure S5 — Effect of Atf7ip depletion on the expression of the Xi-GFP and Xi-linked H2B citrine reporter. This figure parallels Figure 1, and shows the actual fluorescent intensities of the Xi-linked fluorescent reporters upon various treatments. (A) Top: (Left) Schematic of the X chromosomes in female reporter MEFs heterozygous for the CAG-GFP reporter. Due to random XCI, the GFP is on the Xi in approximately half the population. (Right) When in conjunction with the Xist knockout allele, the CAG-GFP reporter is on the Xi in 100% of the cells (XiCAG-H2BCitrineXaΔXist) due to skewing with Xist deletion. Bottom: GFP fluorescence was quantified by flow cytometry in the MEFs depicted above, treated with the indicated conditions for 72 h, and the FACS results are displayed. (B) (i) Schematic of the X chromosomes in female reporter MEFs carrying a heterozygous H2B-Citrine reporter (within the Hprt locus) on the Xi in all cells of the population (XiCAG-H2BCitrineXaΔXist MEFs). (ii) Citrine fluorescence was quantified by flow cytometry in these MEFs treated with the indicated conditions for 72 h. Note siControl knockdown produced significant background citrine fluorescence signal compared to the untreated sample. (iii, iv) FACS plots for data summarized in (ii) with initial cell gating (iii) and citrine-positive cell gating shown (iv). [file 1756-8935-7-12-S5.pdf]

# Supplemental Figure 7

A

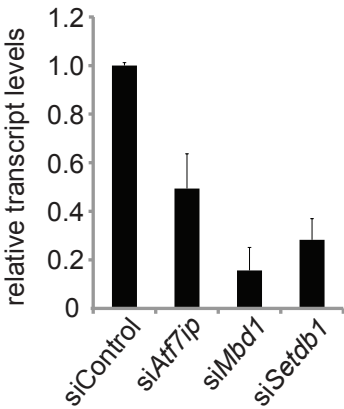

B

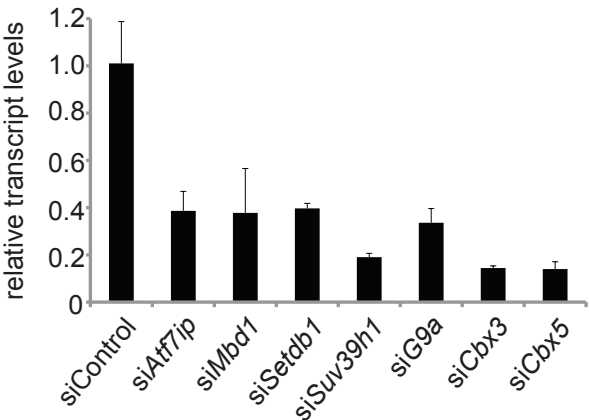

Supplement: Additional file 7: Figure S7 — Confirmation of knockdown of Atf7ip-related factors. This figure supports Figure 4 to show that extent of knockdown of various transcripts by RT-PCR in the same experiments. (A) MEFs were treated with siRNAs targeting the indicated genes or siControl (siGFP) as described in Figure 4 and respective transcript levels were determined by RT-qPCR (that is, 40% of Atf7ip transcription remaining after siAft7ip treatment, 40% of Mbd1 transcript remaining after siMbd1 treatment, and so on). The data were normalized to the siGFP (control) treatment and to Gapdh expression. Error bars indicate one standard deviation from triplicate RT-qPCR measurements in one representative experiment. (B) As in (A), but for the experiment shown in Figure 4C in the untreated condition (without 5-aza-2’-dC or siDnmt1). [file 1756-8935-7-12-S7.pdf]

Supplemental Figure 8

Female MEFs

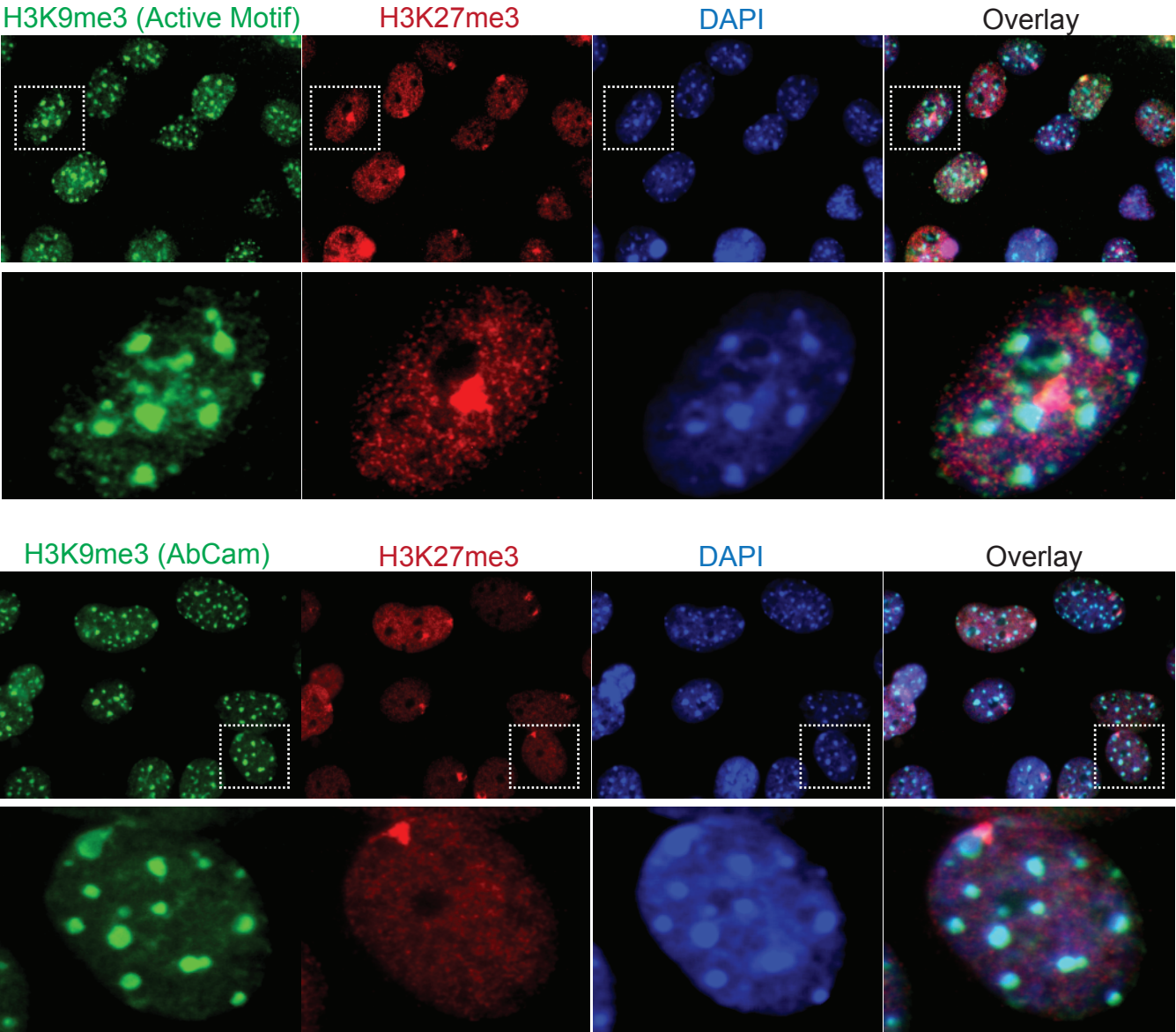

Supplement: Additional file 8: Figure S8 — H3K9me3 localization relative to the H3K27me3 Xi-domain. This figure contains immunostaining images for H3K9me3 relative to H3K27me3 to identify the Xi. Representative immunostaining images of female MEFs for H3K9me3 (green, with two different antibodies termed Active Motif and AbCam) and H3K27me3 (red) with zoomed-in views of a single nucleus. DAPI was used to demarcate the nuclei. [file 1756-8935-7-12-S8.pdf]
