# Supplementary material for: The Mbd1-Atf7ip-Setdb1 pathway contributes to the maintenance of X chromosome inactivation
Source: Epigenetics Chromatin. 2014 Jun 26;7:12. doi: 10.1186/1756-8935-7-12 (PMC4099106; doi:10.1186/1756-8935-7-12)
Supplement: Additional file 6: Figure S6 — Atf7ip does not control the Xist-dependent accumulation of the chromatin regulator ASH2L on the Xi. This figure supports Figure 2 with FISH and immunostaining to demonstrate that Atf7ip knockdown, with or without a low dose 5-aza-2’-dC (0.2 uM), does not change the extent of Xi enrichment/localization of Ash2l. It also shows that rates of Xist RNA coating of the Xi are not affected in cells that display the reactivation of the Xi-linked-GFP reporter. (A) Immunostaining for ASH2L (red) on MEFs 72 h after transfection of siRNAs targeting Atf7ip or control knockdown (targeting GFP), respectively, and treatment with 5-aza-2’-dC (0.2 uM) similar to the experiment described in Figure 2A. Representative images are shown and DAPI was used to demarcate the nucleus. (B) Graph summarizes the proportion of DAPI-stained nuclei (n = 200 per sample) with and Xi-like accumulation of ASH2L for the experiment described in (A) and additional conditions. (C) XiCAG-H2BCitrineXaΔXist MEFs were treated for 72 h with Atf7ip siRNA and 5-aza-2’-dC (0.2 um) or with 5-aza-2’-dC 10.0 uM alone. Given is a representative IF/FISH image for Xist (red) and GFP (green) that depicts a cell with GFP reactivation that contains an Xist RNA coated chromosome. (D) Quantification of Xist RNA coating in GFP + and GFP-negative cells from the experiment described in (C), indicating the Xist RNA coating is maintained in cells displaying reactivation of the Xi-linked GFP. [file 1756-8935-7-12-S6.pdf]

Supplemental Figure 6

A

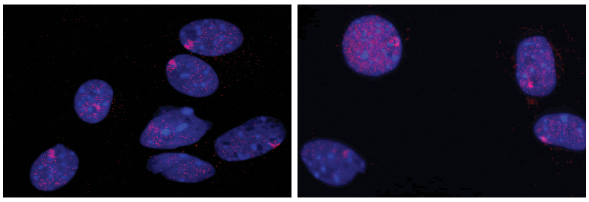

B

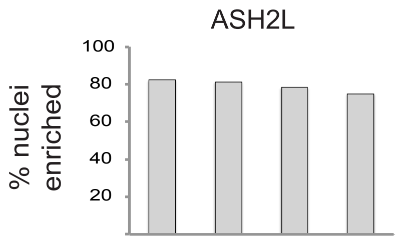

siControl	+	-	+	-
si*Atf7ip*	-	+	-	+
5-aza-2'-dC (0.2uM)	-	-	+	+

C

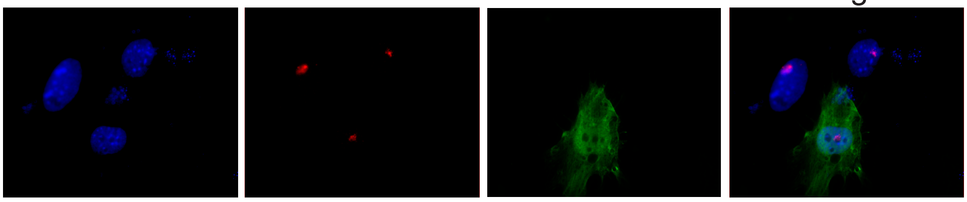

D

		Xist cloud	no *Xist* cloud
si*Atf7ip* + 5-aza-2'-dC (0.2 uM)	GFP+ cells	196	1
	GFP- cells	193	0
5-aza-2'-dC (10 um)	GFP+ cells	203	0
	GFP- cells	202	0
