# Supplementary material for: The Mbd1-Atf7ip-Setdb1 pathway contributes to the maintenance of X chromosome inactivation
Source: Epigenetics Chromatin. 2014 Jun 26;7:12. doi: 10.1186/1756-8935-7-12 (PMC4099106; doi:10.1186/1756-8935-7-12)
Supplement: Additional file 9: Table S1 — Primers for RT-PCR. Primer sequences used for RT-PCR analysis. [file 1756-8935-7-12-S9.doc]

**Supplementary Table 1** (Included in manuscript text)

**Primers for RT-PCR**

Primer sequences used for RT-PCR analysis

| **Name** | **Sequence** |
| --- | --- |
| Gapdh-F | catggccttccgtgttcct |
| Gapdh-R | gcctgcttcaccaccttct |
| Atf7ip-F | atgcaggctcctgccgttcg |
| Atf7ip-R | gggcgtgggggctcattgt |
| Mbd1-F | aactgagctctcccttaaagg |
| Mbd1-R | tgactgctgtccactcctctg |
| Setdb1-F | gcaactcagaacccgtccta |
| Setdb1-R | ataggctgtaggggctccat |
| Dnmt1-F | catgaattcctgcaaacagaa |
| Dnmt1-R | ttgactttagccaggtagcc |
| Suv39h1-F | gctggaaaagatccgaaaaa |
| Suv39h1-R | ctggcggtcgtagatctgg |
| Ehmt2-F | catgtccaaacctagcaacg |
| Ehmt2-R | ccagagttcagcttcctcctt |
| Cbx3-F | ctggaccgtcgtgtagtgaa |
| Cbx3-R | aaattttcttctggttcccaag |
| Cbx5-F | ggaaatccagtttctccaaca |
| Cbx5-R | gctccgatgatcttttctgg |
